# Anticonvulsants and Chromatin-Genes Expression: A Systems Biology Investigation

**DOI:** 10.3389/fnins.2020.591196

**Published:** 2020-11-25

**Authors:** Thayne Woycinck Kowalski, Julia do Amaral Gomes, Mariléa Furtado Feira, Ágata de Vargas Dupont, Mariana Recamonde-Mendoza, Fernanda Sales Luiz Vianna

**Affiliations:** ^1^Postgraduation Program in Genetics and Molecular Biology, Universidade Federal do Rio Grande do Sul (UFRGS), Porto Alegre, Brazil; ^2^Laboratory of Immunobiology and Immunogenetics, Universidade Federal do Rio Grande do Sul (UFRGS), Porto Alegre, Brazil; ^3^National Institute of Population Medical Genetics (INAGEMP), Porto Alegre, Brazil; ^4^Genomic Medicine Laboratory, Hospital de Clínicas de Porto Alegre (HCPA), Porto Alegre, Brazil; ^5^National System of Information on Teratogenic Agents (SIAT), Medical Genetics Service, Hospital de Clínicas de Porto Alegre (HCPA), Porto Alegre, Brazil; ^6^Centro Universitário CESUCA, Cachoeirinha, Brazil; ^7^Bioinformatics Core, Hospital de Clínicas de Porto Alegre (HCPA), Porto Alegre, Brazil; ^8^Institute of Informatics, Universidade Federal do Rio Grande do Sul (UFRGS), Porto Alegre, Brazil

**Keywords:** WGCNA, epigenetics, antiepileptics, teratogen, valproic acid, phenytoin, fetal hydantoin syndrome, fetal valproate syndrome

## Abstract

Embryofetal development is a critical process that needs a strict epigenetic control, however, perturbations in this balance might lead to the occurrence of congenital anomalies. It is known that anticonvulsants potentially affect epigenetics-related genes, however, it is not comprehended whether this unbalance could explain the anticonvulsants-induced fetal syndromes. In the present study, we aimed to evaluate the expression of epigenetics-related genes in valproic acid, carbamazepine, or phenytoin exposure. We selected these three anticonvulsants exposure assays, which used murine or human embryonic stem-cells and were publicly available in genomic databases. We performed a differential gene expression (DGE) and weighted gene co-expression network analysis (WGCNA), focusing on epigenetics-related genes. Few epigenetics genes were differentially expressed in the anticonvulsants’ exposure, however, the WGCNA strategy demonstrated a high enrichment of chromatin remodeling genes for the three drugs. We also identified an association of 46 genes related to Fetal Valproate Syndrome, containing *SMARCA2* and *SMARCA4*, and nine genes to Fetal Hydantoin Syndrome, including *PAX6, NEUROD1*, and *TSHZ1*. The evaluation of stem-cells under drug exposure can bring many insights to understand the drug-induced damage to the embryofetal development. The candidate genes here presented are potential biomarkers that could help in future strategies for the prevention of congenital anomalies.

## Introduction

Embryogenesis is a stepwise controlled process, which requires specific gene expression orchestrated by signaling networks (Rape, 2017). During the embryo development, epigenetics modifications are essential for the correct expression of these highly orchestrated genes, hence enabling the transition from pluripotent stem-cells until its final differentiation state (Rape, 2017; Jambhekar et al., 2019).

The lack of proper chromatin modifications can be lethal during embryogenesis or lead to the occurrence of congenital anomalies (Jambhekar et al., 2019). Embryogenesis failures can be caused by genetic factors or external stimuli, named teratogens (De Santis et al., 2004; Worley et al., 2018). According to epidemiologic studies, it is believed that teratogens cause 10–15% of all the congenital anomalies (Gilbert-Barness, 2010); however, there are many barriers in regard to the proper teratogen identification and understanding of its molecular mechanisms.

Few studies have assessed the potential of a teratogenic drug disrupting epigenetics mechanisms, being these studies restricted especially to alcohol and valproic acid use during pregnancy, and their induced histone hyperacetylation (Tung and Winn, 2010; Gupta et al., 2016; Mazzu-Nascimento et al., 2017). On the other hand, assays in embryonic stem-cells are constantly used in the developmental toxicity field, providing a better comprehension of the drug-induced perturbation in development (Worley et al., 2018; Leigh et al., 2020). These perturbations could be assessed by evaluating how these proteins interact with each other in a biological network, which is systems biology field of research.

From a systems biology perspective, these gene expression perturbations could be identified by network and co-expression analyses, helping to hypothesize which epigenetics mechanisms are teratogen-affected.

Hence, the aim of the present study is to evaluate the effect of anticonvulsant drugs, known for their teratogenic effects, in the expression of epigenetics machinery genes. For its accomplishment, we performed a secondary expression analysis in murine or human embryonic stem-cells (mESC and hESC) exposed to these drugs, and evaluated the results through systems biology strategies, especially the weighted gene correlation network analysis (WGCNA). WGCNA is a consolidated screening method to identify biomarker candidates or therapeutic targets; it associates gene expression and external traits to identify modules of highly correlated genes (Langfelder and Horvath, 2008). Finally, we hypothesized the main genes and epigenetics mechanisms that might be perturbed in these teratogens’ exposure.

## Methods

### Teratogens Selection

Careful literature research was performed to select only drugs with proven teratogenic effects in the human embryo or fetus, and with established animal models. These molecules were named major teratogens and assessed in the DrugBank database to obtain its pharmaceutical class and variant names. Anticonvulsants were the chosen class of study by convenience, according to the availability of genomic expression assays.

### Bioinformatics Analysis

Gene expression studies were obtained through research mechanisms in the ArrayExpress and Gene Expression Omnibus (GEO) databases, using the name of the drugs selected in the search mechanism. Filters were applied to select only exposure studies in murine or human embryonic stem-cells (mESC or hESC). Despite only microarray studies being selected, RNA-seq assays were also considered.

Differential gene expression analysis was performed in the R v.3.6.2, applying robust multiaverage (RMA) normalization, and using the *affy* and *limma* packages. The following comparisons were executed: valproic acid, carbamazepine, phenytoin, methotrexate, and warfarin exposure assays were set against unexposed stem-cells; mESC and hESC selected assays were evaluated separately. All the genes with logFC > 1.5 and adjusted *P*-value for false discovery rate (FDR) < 0.05 were considered upregulated; logFC < −1.5 and the same adjusted *P*-value for FDR were set as parameters for the downregulated genes.

Gene ontologies and Reactome enrichment analysis were also performed in the R v.3.6.2, using the *clusterprofileR* package, considering only significantly enriched ontologies or pathways (FDR < 0.05). Orthologs assessment was performed using the *BiomaRt* package. Only orthologs of high confidence were included, according to the Ensembl Orthology Quality Contro^1^.

Human Phenotype Ontology (HPO) database was assessed in the link^2^. Venn diagrams were performed in the Bioinformatics and Evolutionary Genomics webtool, from the Ghent University^3^.

### Systems Biology Analysis

Weighted gene correlation network analysis (WGCNA) was performed in the R v.3.6.2 with the homonym package; as in DGE analysis, mESC, and hESC datasets were evaluated separately. Data heterogeneity included differences in dose and time of exposure for the mESC studies, hence a consensus analysis was used, as recommended in the WGCNA package tutorials. The 20% probes with larger expression variance were included in the analysis. A thresholding power was set in 12, according to the topology of the data. Default minimum and maximum module sizes were used, comprising of at least 30 and maximum of 3,000 genes per module. Gene significance was set in 0.1. This measure helps to obtain the biologically relevant genes. We selected 0.1 as a threshold value because this is an exploratory study, hence we wanted to collect all the biologically relevant genes. Further phenotype-associated genes were still to be filtered, what would also help to reduce any noise (non-relevant genes).

More information about the WGCNA parameters can be encountered in the tutorials provided by the developers^4^.

Protein-protein interaction networks were generated with the STRING v.11 database webtool, comprising only query proteins, and with a minimum required interaction score of 0.4 (default). The confidence score is a probability that evaluates whether the proteins are included in the same metabolic pathway (von Mering et al., 2005). The medium score we selected might include false positives, therefore, we filtered for experimental data only, to exclude computational predicted interactions. Further network statistics were performed in the Cytoscape v.3.7.2. Considering the size of the network, we performed global centrality analysis, evaluating betweenness centrality as the size and closeness centrality as the color of the nodes. The DyNet v. 1.0.0 Cytoscape application was used for network comparison, using a *prefuse force directed layout* in the network combinations for the WGCNA results and HPO data for the teratogenic syndromes.

## Results

### Teratogens, Expression Datasets Selection, and Epigenetics Genes

We searched for gene expression studies in stem-cells exposed to 28 different teratogens. After a careful evaluation, the anticonvulsants valproic acid, carbamazepine, and phenytoin were chosen for expression and systems biology analysis; the three drugs are folic acid antagonists (Matok et al., 2009). For comparison purposes, methotrexate and warfarin were selected. Methotrexate is an antineoplastic agent and a folic acid antagonist, whilst warfarin is an anticoagulant (De Santis et al., 2004). Supplementary Table 1 comprises the phenotypical spectrum of the embryopathies induced by the drug selected.

Four studies were selected for gene expression and systems biology analysis: E-MTAB-300, E-TABM-1205, and E-TABM-1216 (van Dartel et al., 2011; Theunissen et al., 2012, 2013), from ArrayExpress database (European Bioinformatics Institute, EBI), and GSE64123 (Schulpen et al., 2015) from the Gene Expression Omnibus (GEO) database (National Center of Biotechnology and Information, NCBI). The studies comprised assays evaluating valproic acid (*n* = 32), carbamazepine (*n* = 24), and phenytoin (*n* = 16) exposure in mESC, being all performed in the same platform, of the same laboratory. For comparison purposes, methotrexate (*n* = 8), warfarin (*n* = 8), and non-exposed cells (*n* = 13) were also used in the analysis. Separately, one assay of valproic acid (*n* = 28) or carbamazepine (*n* = 26) in hESC was evaluated and compared to unexposed cells (*n* = 27). The studies selected were all microarray assays from the Affymetrix platforms (Thermo Fisher Scientific, United States). Full characteristics of the assays are available in the ArrayExpress and GEO databases.

In this study, we aimed to focus only in the expression effects on the epigenetics machinery genes. Hence, we performed a Gene Ontology (GO) research, to select all the genes that might be relevant in this scenario. We encountered 593 ontologies related to epigenetics mechanisms (Supplementary Table 2) that were used to filter the epigenetics genes after the DGE and WGCNA analyses were completed. This selection provided 2,091 *Homo sapiens* genes and 1,918 *Mus musculus* genes (Supplementary Table 3).

**FIGURE 1:**
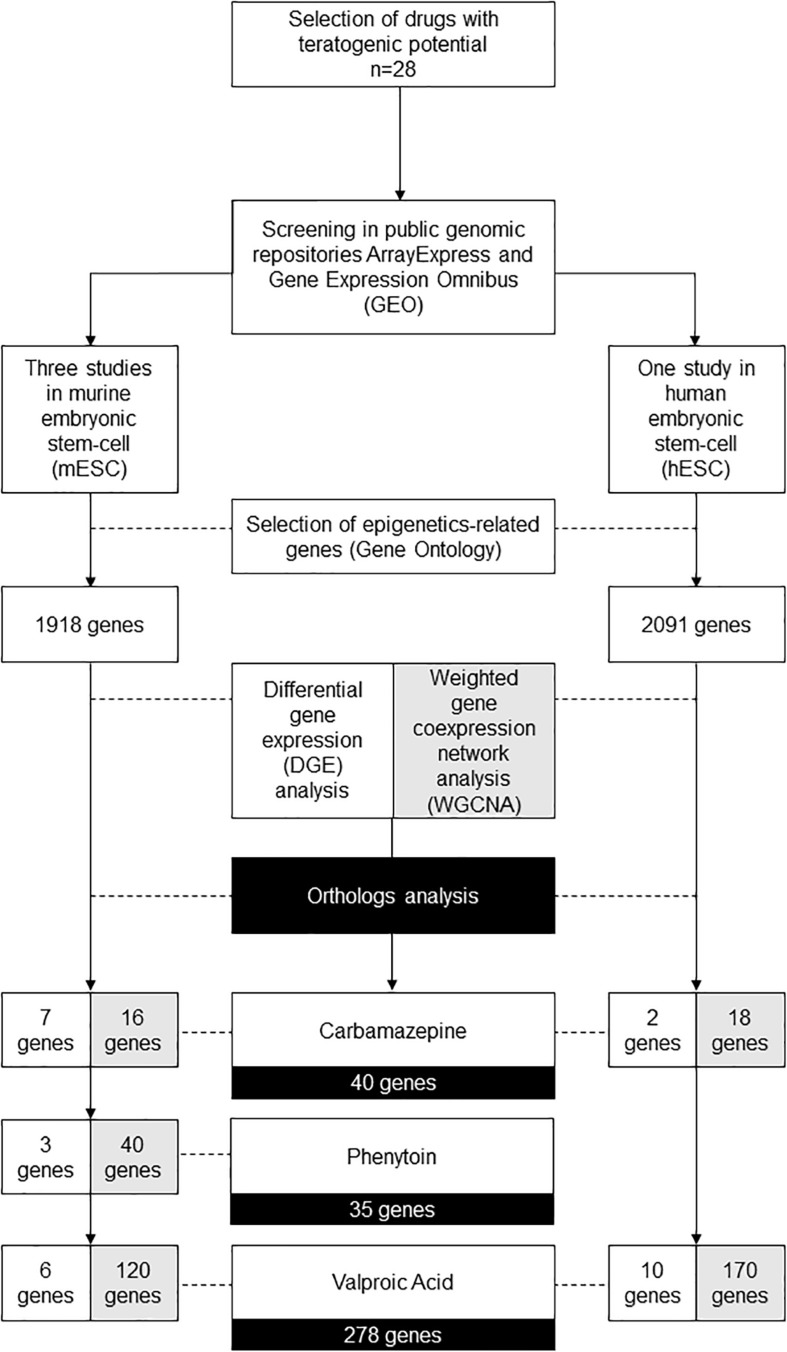
Diagram demonstrating the gene filters for each bioinformatic and systems biology analysis performed.

The diagram available in Figure 1 demonstrates the gene filters applied in the following bioinformatics and systems biology analysis.

### Differential Gene Expression Analysis

Despite the epigenetics machinery genes being restrictedly regulated, we evaluated whether the selected teratogens could influence in their gene expression. We evaluated each dataset separated by concentration, time of exposure, and teratogen. Similar results to the ones already published by the group that performed the primary analysis in these datasets were encountered (Theunissen et al., 2012, 2013; Schulpen et al., 2015). Hence, we do not present it. Then, we joined the samples of cells exposed to different concentrations in different time-points for a same drug. This union was especially with the intention of evaluating which epigenetics-related genes are deregulated, independently of the concentration and time of exposure. We compared the differentially expressed genes to the epigenetics-related ones selected with the GO analysis.

Using the epigenetics machinery genes filter for the mESC assays, only the genes *Tshz1* and *Pax6* were upregulated in phenytoin or valproic acid exposure; however, both were downregulated in carbamazepine, methotrexate, or warfarin exposure. An opposite effect was seen for *Eomes* gene, which was downregulated when in exposure of valproic acid or phenytoin, and upregulated after carbamazepine, warfarin, or methotrexate treatment. *Lef1* and *Meis1* also had discordant results between the teratogens. Supplementary Table 4 comprises a complete list of the logFC values, GO, and these genes’ main functions, which were identified as mainly related to chromatin binding (GO:0003682).

When evaluating the hESC study, only one gene related to epigenetics mechanisms was downregulated in valproic acid exposure, and eight were upregulated. In carbamazepine treatment, only two downregulated genes were identified. None were in common between both drugs. Supplementary Table 5 comprises the main characteristics for the genes differentially expressed in the hESC exposure assay.

In summary, few epigenetics-related genes were differentially expressed in the anticonvulsants’ exposure. However, it was not possible to confirm whether these genes correlated expression was also unaffected. Hence, to perform a co-expression evaluation, we proceeded with the WGCNA analysis.

### Weighted Gene Correlation Network Analysis (WGCNA)

WGCNA analysis was applied to better comprehend which genes of the epigenetics machinery are mostly affected by the chosen drugs.

According to its developers, WGCNA can only be applied in sets with a high number of samples (preferentially above 15), hence methotrexate and warfarin were excluded from this analysis. All the samples used were of the fourth day after exposure to different drug concentrations (Supplementary Table 6), and they were all retrieved from E-TABM-1205 and E-TABM-1216. As in the DGE analysis, we wanted to verify the variable co-expression when using different concentrations of these drugs. According to the suggestions given by the WGCNA package developers, a variance filter was applied to select only the genes with higher deviation from the genes’ mean expression.

Supplementary Figure 1 graphically represents the filters applied in the mESC datasets, until we obtained a final list of genes related to epigenetic mechanisms. First, a variance filter was applied in mESC assays and provided 7,588 probes for WGCNA analysis. The following modules were encountered for each anticonvulsant: eight for carbamazepine, 13 for phenytoin, and nine for valproic acid. Second, we evaluated the highly significant modules, considering only the modules with a gene ratio of at least 0.1; this cutoff implies all the modules selected had a high ratio of clustered genes that might be associated to phenotypical traits. Hence, of the 7,588 probes with greatest variance, it was possible to identify one highly significant module for valproic acid, containing 1,124 genes, and two modules for carbamazepine and phenytoin each. Carbamazepine modules contained 113 and 50 genes, whilst phenytoin modules had both 227 genes included. Cluster dendrogram is available in Supplementary Figure 2.

Finally, we evaluated the genes enriched in these modules, filtering only for the ones that were related with epigenetics mechanisms, according to the list we previously obtained, which is available in Supplementary Table 3. In regard to this GO analysis, 120 probes of the valproic acid significant module were related to epigenetics mechanisms, 40 from the two significant phenytoin modules and 16 from the carbamazepine ones had epigenetics role. *Prdm14* was the hub gene for one of the significant modules presented in phenytoin exposure; the other modules did not have epigenetics gene as their main or most connected hub.

The same process was applied in the hESC exposure assay, and is graphically represented in Supplementary Figure 3. First, WGCNA analysis was performed in the 10,943 probes with greater variance. The dendrogram with the hierarchical clustering method applied by WGCNA is available in Supplementary Figure 4. Second, there were 217 and 3,776 genes presented in significant modules for carbamazepine and valproic acid, respectively. These genes were presented in three significant modules of the 13 identified, when evaluating the cells with carbamazepine exposure, and four significant modules in eight were identified, when in valproic acid treatment. Finally, we filtered for the genes related to epigenetics mechanisms, and obtained a list of eighteen probes of the carbamazepine assay, and 170 probes of the valproic acid treatment. One of the significant modules for valproic acid has an epigenetics machinery gene as its main hub: *ZMYND11*.

In summary, at the end of WGCNA, it is possible to identify modules of highly clustered genes, that might have an association with phenotypical traits, or even experimental conditions. For all the modules identified, we selected only the epigenetics related genes. From this filtered list, we performed an ortholog analysis to obtain a final list of genes for the three drugs evaluated, also comprising the genes identified in differential expression analysis. Hence, at the end of these analyses, 278 genes for valproic acid, 40 genes for carbamazepine, and 35 genes for phenytoin (Figure 1). The complete list of the genes is available in Supplementary Table 7.

After achieving a final list of genes, we aimed to evaluate its association to the clinical traits by performing network statistics analysis and evaluating gene-phenotype association.

### Network Statistics

Besides the WGCNA analysis, other network statistics were applied to evaluate the main characteristics of the genes identified in the previous step. Our aim was to verify whether the epigenetics mechanisms deregulated by the different teratogens were similar for each drug.

**FIGURE 2:**
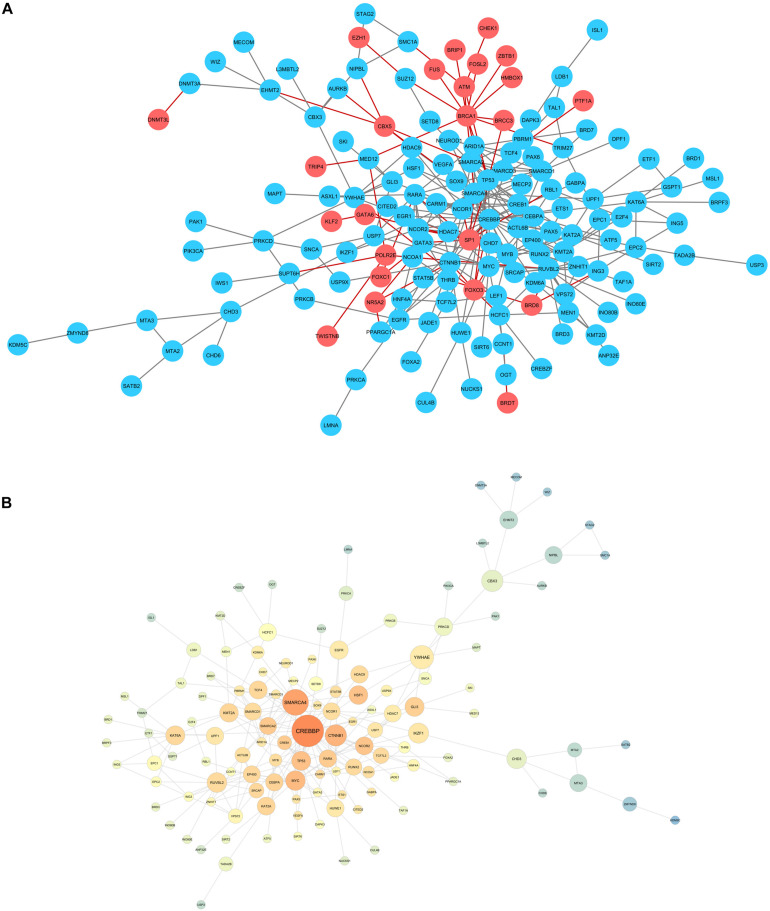
**(A)** Network for the candidate genes encountered for valproic acid (blue), compared to the genes obtained in carbamazepine and phenytoin evaluations. **(B)** Network statistics for valproic acid selected genes. Warm colors: high closeness centrality score. Node size: big nodes for genes with high betweenness centrality score.

When evaluating carbamazepine and phenytoin candidate genes, it was not possible to assemble a protein-protein interaction network, probably because of the small number of genes selected. Hence, a valproic acid network was generated, and compared to a network containing all the genes selected for the three drugs (Figure 2A).

Network statistics analysis was also performed for valproic acid, evaluating betweenness and closeness centrality to identify the genes with bigger information flow (Figure 2B). *CREBBP* was the gene with the bigger betweenness centrality, although we highlight the chromatin remodeling genes (*SMARCA4, SMARCA2, SMARCD1, and SMARCD3*) in the center of the network.

**FIGURE 3:**
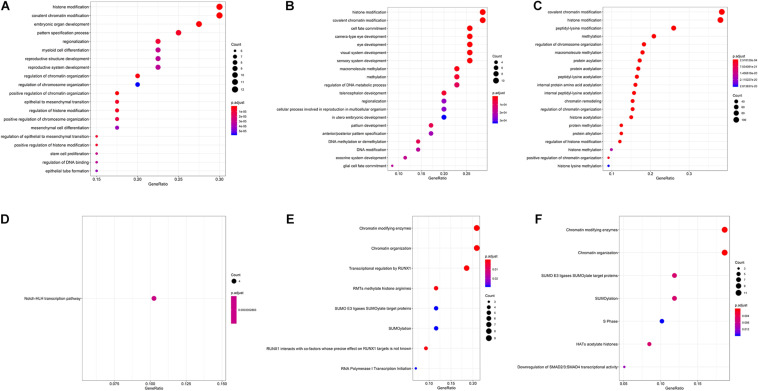
Gene ontologies enrichment for carbamazepine **(A)**, phenytoin **(B)**, and valproic acid **(C)** selected genes, and Reactome database enriched pathways for carbamazepine **(D)**, phenytoin **(E)**, and valproic acid **(F)** drugs.

To verify what are the main epigenetics mechanisms associated to the selected genes, we performed another GO analysis, and evaluated the main pathways they were included, according to the Reactome database. Significantly enriched GO and Reactome pathways can be assessed in Figures 3A–F. Besides epigenetics pathway ontologies, many embryo development ontologies were also enriched.

Therefore, after network statistics evaluation, chromatin remodeling was the main epigenetic mechanism suggested to be affected in the anticonvulsants’ exposure. The following analysis was intended to understand if these genes might have a role in the phenotypical spectrum of these teratogens-induced embryopathies.

### Gene-Phenotype Associations

Systems biology analyses provided several epigenetic genes potentially deregulated by the anticonvulsant drugs here evaluated. To better comprehend how these genes could also influence in the teratogenic potential of these drugs, we evaluated the phenotypical spectrum of the embryopathies caused by these teratogens, as comprised in Supplementary Table 1.

To assess the gene-phenotype association, Human Phenotype Ontology (HPO) database was used. Carbamazepine teratogenesis is not registered in this repository, however Fetal Valproate Syndrome (ORPHA 1906) and Fetal Hydantoin Syndrome (ORPHA 1912) phenotypes caused by valproic acid and phenytoin, respectively, were annotated.

**FIGURE 4:**
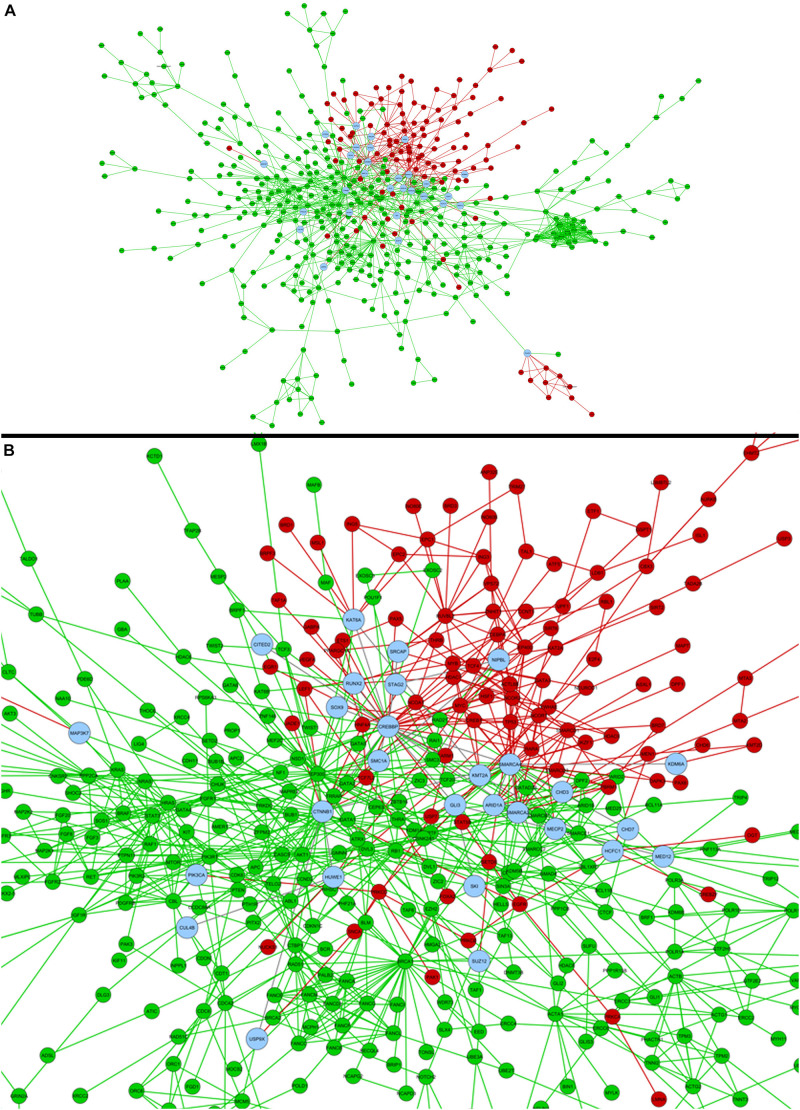
**(A)** Comparison of valproic acid candidate genes obtained in the present study (red) and former HPO database registered genes for Fetal Valproate Syndrome (green). Common genes between both strategies are represented in blue, which can be better visualized in the zoom in **(B)**.

A comparison between the genes associated for each phenotype was executed against the list of candidate genes obtained through the systems biology analyses here executed. Valproic acid evaluation provided 46 genes in common, between HPO and 278 epigenetic genes we selected, including chromatin remodeling genes *SMARCA2* and *SMARCA4*, and *CREBBP*, with the bigger value of betweenness centrality in the network statistical analysis (Figure 4). *KMT2A* and *SMC1A* were associated to five phenotypes, each. For phenytoin, of the 35 selected genes, nine were registered in the HPO database, including genes identified in the differential expression analyses, such as *PAX6*, *NEUROD1*, and *TSHZ1*. *PAX6* was associated to eight phenotypes. The complete list of genes associated to HPO phenotypes is available in Supplementary Table 8.

## Discussion

The present study aimed to investigate the effect of valproic acid, carbamazepine, and phenytoin in the expression of genes with epigenetic-related mechanisms. This objective was accomplished by performing a careful systems biology evaluation using assays available in public genomic repositories and suggesting possible candidate genes for future researches.

With the differential gene expression analysis combined with WGCNA, 278 epigenetics genes were associated to valproic acid exposure, 40 to carbamazepine, and 35 to phenytoin. Combining HPO database evaluation, 46 epigenetics-related genes were associated to Fetal Valproate Syndrome and nine to Fetal Hydantoin Syndrome. The elevated number of “chromatin remodeling” enriched Gene Ontologies suggest this mechanism as a relevant mechanism for teratogenic disruption, which must be further evaluated in developmental toxicity assays.

The anticonvulsants here evaluated are known as neuroteratogens, because they might affect brain development, especially in second trimester, the period of continuous growth and maturation of the human brain (Ornoy, 2006; Tomson et al., 2019). Fetus exposed to these drugs—carbamazepine, phenytoin, and valproic acid—might present major congenital anomalies (especially craniofacial ones) and development delay related to this exposure, even in monotherapy (Ornoy, 2006; Tomson et al., 2019). Despite the similarities regarding the therapeutic effects, the dysmorphic features for each syndrome is very specific. This pattern might be explained by distinct molecular mechanisms for each drug, which may cause a dissimilar biological perturbation in the brain development. Hence, the main purpose of performing the WGCNA analysis was to identify these perturbations, by assessing potential biomarkers and candidate genes that might help in the comprehension of the phenotypical spectrum for each syndrome. This is the main goal of the WGCNA package, as proposed by its developers (Langfelder and Horvath, 2008).

It is well established that maternal exposure to different agents trigger epigenetic mechanisms, altering the gene expression and, consequently, impairing the embryofetal development (Salilew-Wondim et al., 2014). Despite that, few studies have evaluated the potential epigenetic disruption led by a teratogen exposure; recreational drugs such as alcohol and tobacco have been mainly assessed, as well as maternal infections (Banik et al., 2017; Chang et al., 2019). However, few drugs have been evaluated about these same mechanisms. It is estimated 90% of the women take at least one medication during pregnancy (Mitchell et al., 2011). Hence, the present study is an exploratory assay which attempts to fill these gaps in the understanding of teratogenesis and epigenetics linkage.

Together with antineoplastic agents, valproic acid has been one of the few drugs with a proposed epigenetic mechanism of teratogenesis (Mazzu-Nascimento et al., 2017). Valproic acid is a potent inhibitor of the histone deacetylase enzymes (HDAC), hence promoting an increased level of these proteins’ acetylation. Other studies have demonstrated valproic acid also demethylates DNA (Milutinovic et al., 2007), what might be linked to its role as a folic acid antagonist. Here, we identified not only valproic acid association to DNA methylation and histone acetylation mechanisms, but also to chromatin remodeling genes. These candidate genes proposed are potentially important for the understanding not only of Fetal Valproate Syndrome, but also of other neurodevelopment disorders. Valproic acid is a known inducer of autism in rodent models (Nicolini and Fahnestock, 2018). Some researches point to histone acetylation alterations as associated to neurogenesis impairment, leading to postnatal autistic-like behaviors (Contestabile and Sintoni, 2013), although other epigenetics mechanisms must also be further investigated.

The ortholog analysis also brought potential candidates for Fetal Hydantoin Syndrome. Despite teratogenesis outcomes being variable between species, the mechanisms of many congenital anomalies have been suggested after extensive animal model assays (Shahbazi and Zernicka-Goetz, 2018). The candidates for phenytoin embryopathy, however, must be carefully evaluated before being extrapolated. Some of the genes we encountered have stablished roles in epigenetics mechanisms. *Prdm14* was the hub gene in WGCNA analysis in mESC cells with phenytoin exposure. The encoded protein is a transcription regulator with a consolidated role in pluripotency and epigenome establishment, especially wide DNA demethylation, in mESC. In hESC, its role is more associated with pluripotency regulation (Nakaki and Saitou, 2014). This gene has not been registered in HPO, neither in the Online Mendelian Inheritance in Man (OMIM) database; therefore, its role in genetic syndromes or teratogenic-induced embryopathies must be further investigated.

For the WGCNA analysis in hESC, one of the main hubs identified was *ZMYND11*. It is responsible for the reading of the histone H3K36 trimethylation, specific for H3.3, a H3 histone variant (Wen et al., 2014). This mechanism has been evaluated in tumor suppression, but not in embryo development (Wen et al., 2014). Nevertheless, *ZMYND11* is registered in HPO, being associated with intellectual disability and facial dysmorphisms. These are common phenotypes in Mendelian disorders related to the epigenetics machinery genes (Bjornsson, 2015). Teratogenic-induced malformations are phenotypically similar to the ones caused by genetic syndromes, named phenocopies (Cassina et al., 2017). Therefore, genes like *ZMYND11*, already associated to genetic syndromes, are good candidates for the understanding of teratogenic embryopathies.

Our study lacks proper validation of the candidate genes proposed. Nevertheless, we highlight this as an exploratory research that used only previously validated experimental data for the systems biology and bioinformatics analysis. Much time and effort can be saved by conducting previous hypotheses-generator studies, targeting for biologically relevant genes or proteins (Kowalski et al., 2019). Systems biology is a feasible area for these strategies, due to its integrative and holistic characteristic (Hood et al., 2008; Le Novère, 2015). Notwithstanding, valproic acid was not only the target of our study, but also a marker of the analysis. The identification of histone acetylation genes included in significant modules for valproic acid exposure, was an incidental marker that the method was correctly applied in this investigation.

One of the strong points of our study is that many chromatin remodelers were indicated as good candidates for Fetal Valproate Syndrome understanding, including for genes of the *SMARCA* subgroup, part of the SWI1/SNF1 family (Pulice and Kadoch, 2016). These complexes enable chromatin accessibility by providing a dynamic control in an ATP-dependent mechanism (Clapier and Cairns, 2009). *SMARCA-*deficiencies are associated to several malignancies and birth defects; its homozygous loss lead to embryo lethality (Pulice and Kadoch, 2016). Valproic acid is known to alter the expression of *SMARCA4* and *SMARCD1* in neuroblastoma cells (Hu et al., 2020), and *SMARCA* genes are suggested as members of the neurogenic transcriptional network control (Higgins et al., 2019). Hence, valproate-induced perturbances in *SMARCA* genes might be sufficiently disruptive to explain Fetal Valproate Syndrome.

Finally, it has been hypothesized the understanding of epigenetic mechanisms of teratogenesis could be later used in primary prevention of congenital anomalies (Martínez-Frías, 2010). To its accomplishment, it is necessary to better comprehend these drugs’ effects in chromatin during embryo development. This study was a first step in this investigation, which in future might help counseling many women who need to use these drugs during pregnancy.

## Data Availability Statement

All data studied is available in the ArrayExpress database (European Bioinformatics Institute, EBI), under codes: E-MTAB-300, E-TABM-1205, and E-TABM-1216 (Theunissen et al., 2012, 2013), and in Gene Expression Omnibus (GEO) database (National Center of Biotechnology and Information, NCBI), series GSE64123 (Schulpen et al., 2015).

## Ethics Statement

All the genomic data used in the present research are publicly available in the ArrayExpress and Gene Expression Omnibus databases. There was no use of privileged information or data in this study.

## Author Contributions

TK contributed in devising the concept, designing and conducting the analyses, and writing the manuscript. JG contributed in devising the concept, designing the experiment, and performing the analyses. MF and ÁD contributed in performing the analyses. MR-M contributed in devising and supervising the analyses. FV contributed in devising the concept, designing the experiments, supervising the analyses, and correcting the manuscript. All authors discussed the results and contributed scientifically to the manuscript.

## Conflict of Interest

The authors declare that the research was conducted in the absence of any commercial or financial relationships that could be construed as a potential conflict of interest.

## Footnotes

1 https://www.ensembl.org/info/genome/compara/Ortholog_qc_manual.html

2 https://hpo.jax.org/app/

3 http://bioinformatics.psb.ugent.be/webtools/Venn/

4 https://horvath.genetics.ucla.edu/html/CoexpressionNetwork/Rpackages/WGCNA
