# Supplementary material for: Anticonvulsants and Chromatin-Genes Expression: A Systems Biology Investigation
Source: Front Neurosci. 2020 Nov 25;14:591196. doi: 10.3389/fnins.2020.591196 (PMC7732676; doi:10.3389/fnins.2020.591196)
Supplement: Supplementary file 1 [file Data_Sheet_1.pdf]

Supplementary Material

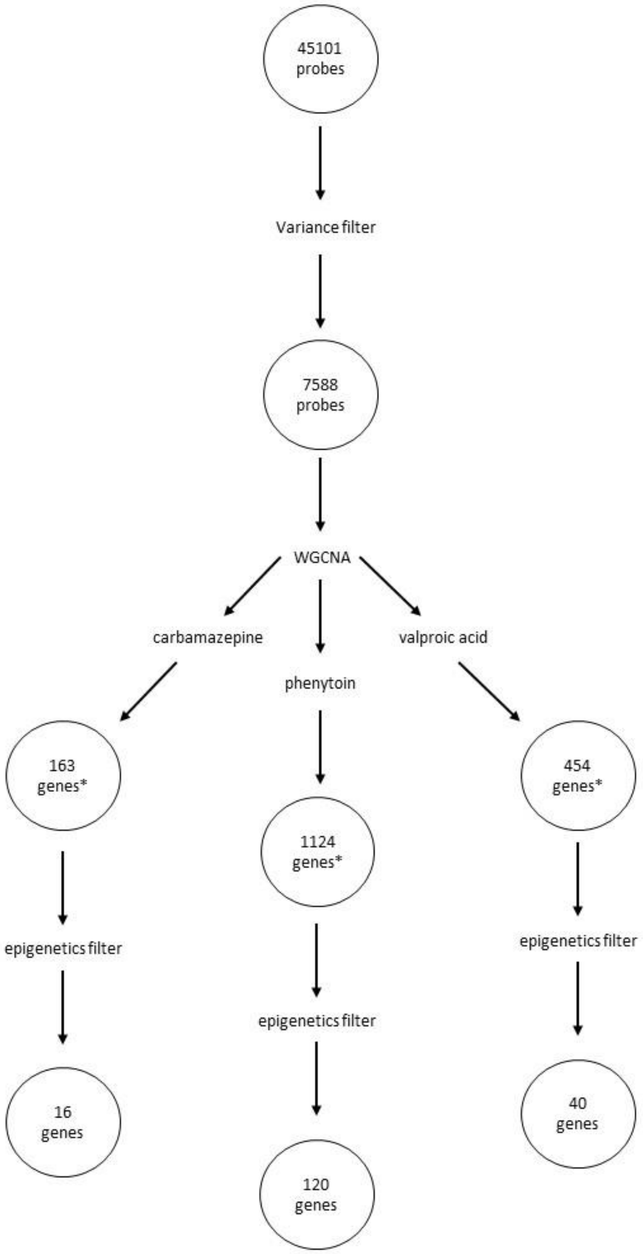

Supplementary Figure 1. Stepwise gene filtering in WGCNA analysis for mouse embryonic stem-cells (mESC) studies

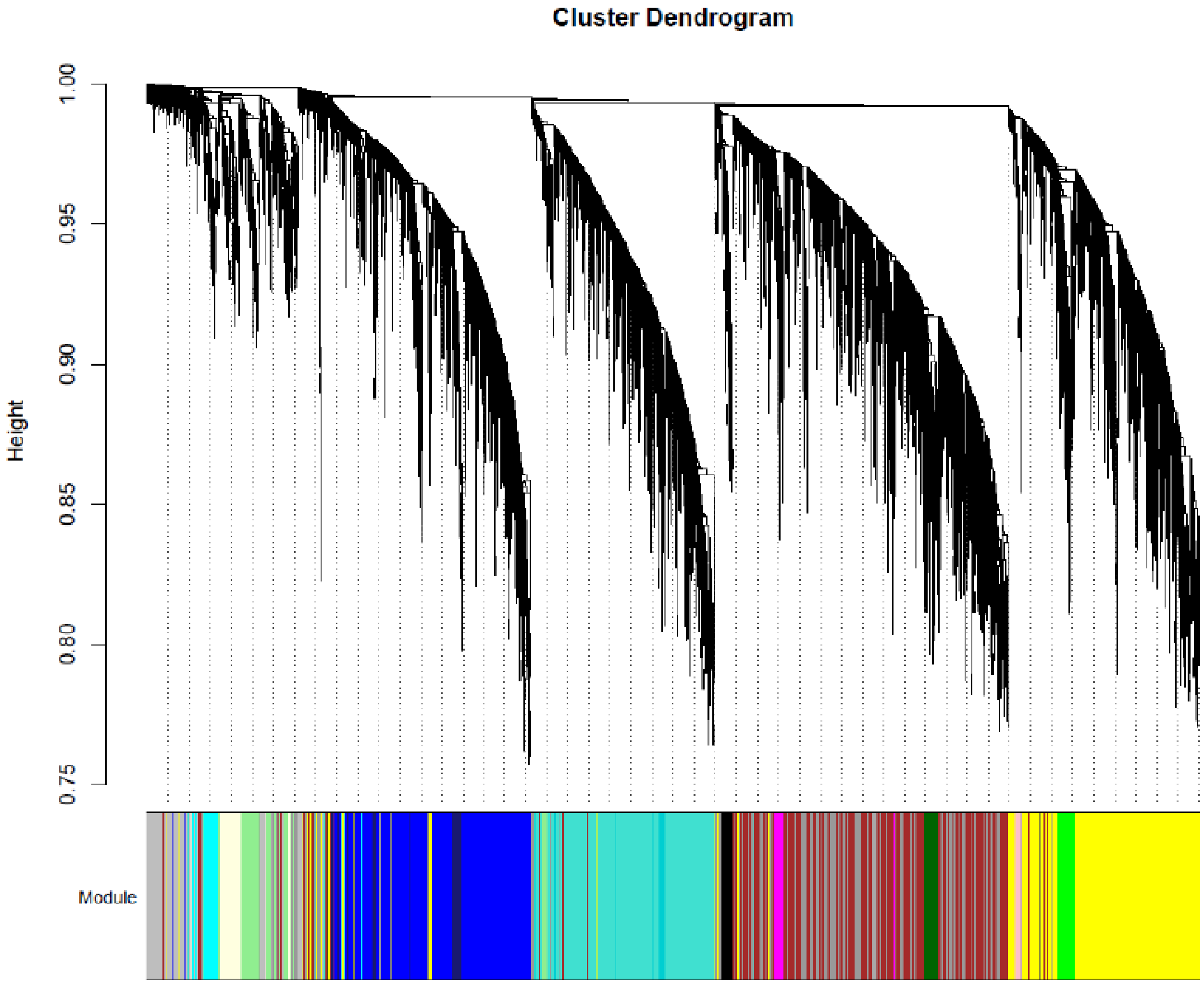

Supplementary Figure 2. Consensus dendrogram for murine embryonic stem-cells (mESC) assays

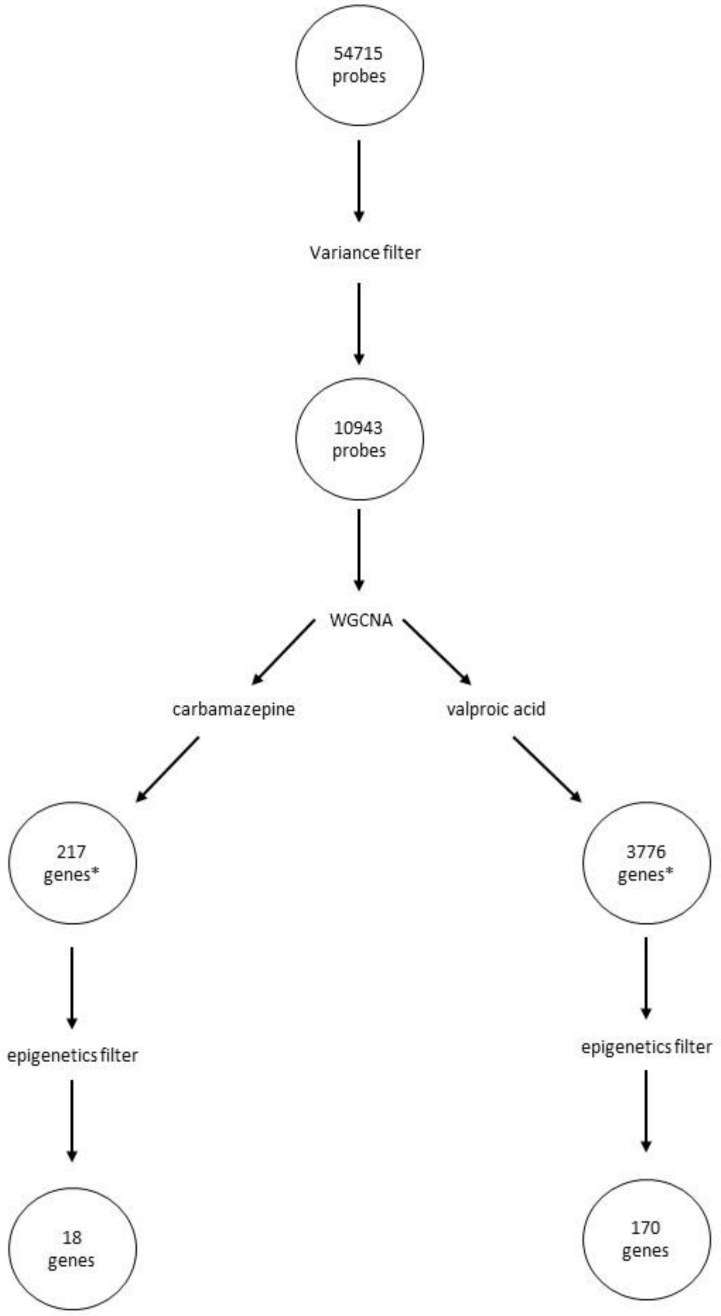

Supplementary Figure 3. Stepwise gene filtering in WGCNA analysis for human embryonic stem-cells (hESC) study

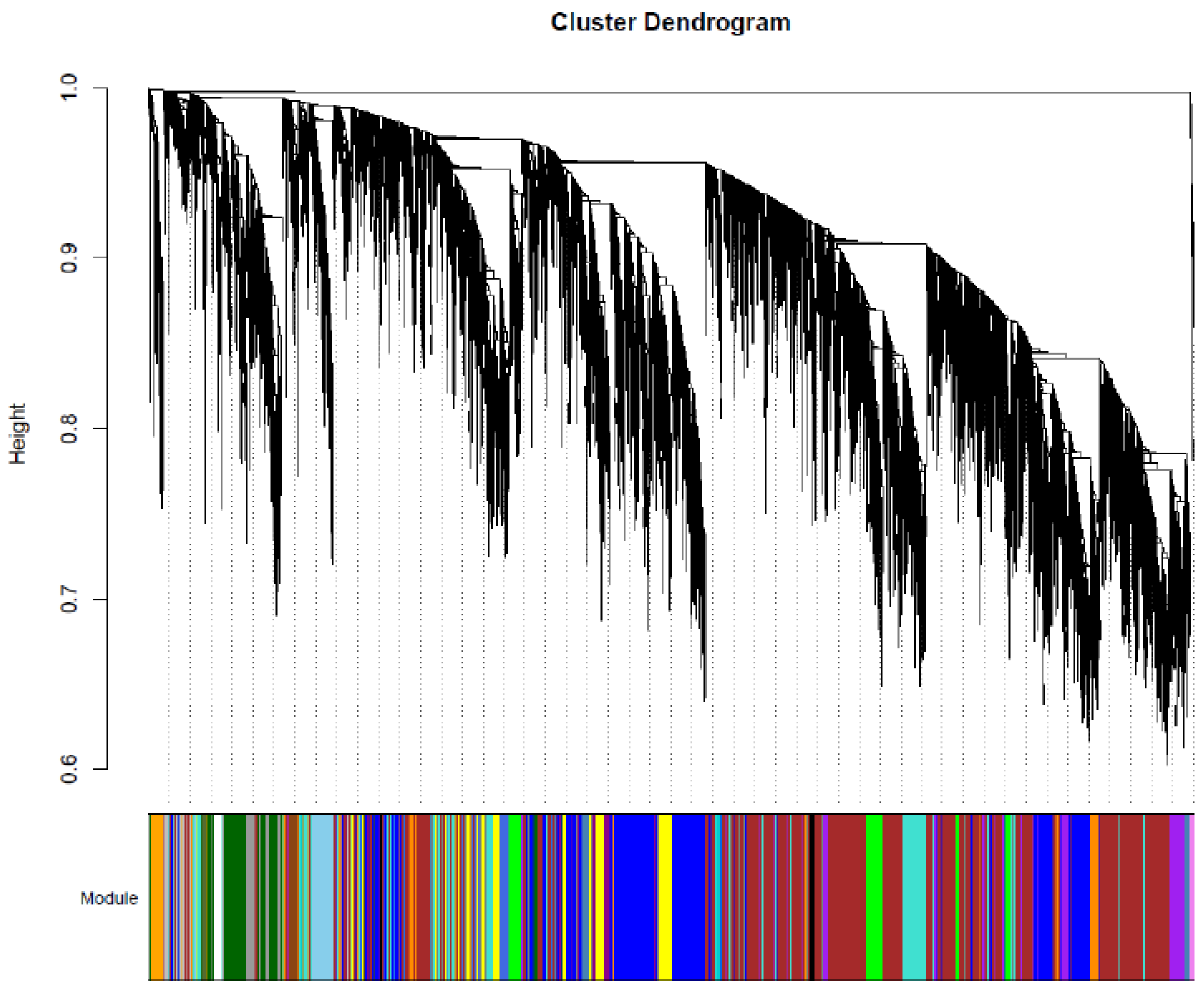

Supplementary Figure 4. Consensus dendrogram for human embryonic stem-cells (hESC) assays

Supplementary Table 1. Clinical features of antiepileptic drugs induced teratogenic syndromes

Drug	Name of the Syndrome	Congenital Anomalies	Dose Dependency
Carbamazepine	ND	spina bifida<br>cardiovascular anomalies<br>cleft lip and cleft palate<br>skeletal anomalies<br>brain anomalies<br>developmental delay	Unknown
Phenytoin	Fetal Hydantoin Syndrome (OMIM 617955)	heart defects<br>facial clefts<br>digital hypoplasia<br>short nose<br>low nasal bridge<br>hypertelorism<br>abnormal ears<br>wide mouth<br>hypoplasia of distal phalanges and nails<br>finger-like thumbs<br>short or webbed neck<br>abnormalities of growth<br>delayed motor development<br>developmental delay<br>intrauterine growth retardation<br>microcephaly	Unknown
Valproic Acid	Fetal Valproate Syndrome (OMIM 609442)	neural tube defects<br>preaxial limb defects<br>heart defects<br>liver defects<br>oral clefts<br>blepharoptosis<br>nasolacrimal duct obstruction<br>craniostenosis<br>hypospadia<br>developmental delay<br>autism<br>Microcephaly	Above 1000mg/day

Supplementary Table 2. Gene Ontologies related to epigenetics mechanisms

GO	Description
GO:0000183	chromatin silencing at rDNA
GO:0000412	histone peptidyl-prolyl isomerization
GO:0000414	regulation of histone H3-K36 methylation
GO:0000415	negative regulation of histone H3-K36 methylation
GO:0000416	positive regulation of histone H3-K36 methylation
GO:0000451	rRNA 2'-O-methylation
GO:0000452	snoRNA guided rRNA 2'-O-methylation
GO:0000453	enzyme-directed rRNA 2'-O-methylation
GO:0001207	histone displacement
GO:0001208	histone H2A-H2B dimer displacement
GO:0001301	progressive alteration of chromatin involved in cell aging
GO:0001304	progressive alteration of chromatin involved in replicative cell aging
GO:0001305	progressive alteration of chromatin involved in chronological cell aging
GO:0001308	negative regulation of chromatin silencing involved in replicative cell aging
GO:0001510	RNA methylation
GO:0001672	regulation of chromatin assembly or disassembly
GO:0002127	tRNA wobble base cytosine methylation
GO:0002128	tRNA nucleoside ribose methylation
GO:0002129	wobble position guanine ribose methylation
GO:0002130	wobble position ribose methylation
GO:0002131	wobble position cytosine ribose methylation
GO:0002132	wobble position uridine ribose methylation
GO:0002938	tRNA guanine ribose methylation
GO:0002939	tRNA N1-guanine methylation
GO:0002940	tRNA N2-guanine methylation
GO:0002946	tRNA C5-cytosine methylation
GO:0003682	chromatin binding
GO:0004402	histone acetyltransferase activity
GO:0004407	histone deacetylase activity
GO:0006306	DNA methylation
GO:0006325	chromatin organization
GO:0006333	chromatin assembly or disassembly
GO:0006338	chromatin remodeling
GO:0006342	chromatin silencing
GO:0006343	establishment of chromatin silencing
GO:0006344	maintenance of chromatin silencing
GO:0006346	methylation-dependent chromatin silencing
GO:0006348	chromatin silencing at telomere
GO:0006479	protein methylation

GO:0006480	N-terminal protein amino acid methylation
GO:0006481	C-terminal protein methylation
GO:0006482	protein demethylation
GO:0008334	histone mRNA metabolic process
GO:0008469	histone-arginine N-methyltransferase activity
GO:0010216	maintenance of DNA methylation
GO:0010390	histone monoubiquitination
GO:0010424	DNA methylation on cytosine within a CG sequence
GO:0010425	DNA methylation on cytosine within a CNG sequence
GO:0010426	DNA methylation on cytosine within a CHH sequence
GO:0010452	histone H3-K36 methylation
GO:0010484	H3 histone acetyltransferase activity
GO:0010485	H4 histone acetyltransferase activity
GO:0010847	regulation of chromatin assembly
GO:0010848	regulation of chromatin disassembly
GO:0010964	regulation of chromatin silencing by small RNA
GO:0016569	covalent chromatin modification
GO:0016570	histone modification
GO:0016571	histone methylation
GO:0016572	histone phosphorylation
GO:0016573	histone acetylation
GO:0016574	histone ubiquitination
GO:0016575	histone deacetylation
GO:0016576	histone dephosphorylation
GO:0016577	histone demethylation
GO:0016578	histone deubiquitination
GO:0017136	NAD-dependent histone deacetylase activity
GO:0018011	N-terminal peptidyl-alanine methylation
GO:0018012	N-terminal peptidyl-alanine trimethylation
GO:0018013	N-terminal peptidyl-glycine methylation
GO:0018014	N-terminal peptidyl-methionine methylation
GO:0018015	N-terminal peptidyl-phenylalanine methylation
GO:0018016	N-terminal peptidyl-proline dimethylation
GO:0018019	N-terminal peptidyl-glutamine methylation
GO:0018020	peptidyl-glutamic acid methylation
GO:0018021	peptidyl-histidine methylation
GO:0018022	peptidyl-lysine methylation
GO:0018023	peptidyl-lysine trimethylation
GO:0018024	histone-lysine N-methyltransferase activity
GO:0018026	peptidyl-lysine monomethylation
GO:0018027	peptidyl-lysine dimethylation
GO:0018110	histone arginine kinase activity
GO:0018125	peptidyl-cysteine methylation
GO:0018181	peptidyl-arginine C5-methylation
GO:0018216	peptidyl-arginine methylation
GO:0018361	peptidyl-glutamine 2-methylation

GO:0018364	peptidyl-glutamine methylation
GO:0019701	peptidyl-arginine N5-methylation
GO:0019710	peptidyl-asparagine methylation
GO:0019716	N-terminal peptidyl-alanine monomethylation
GO:0019918	peptidyl-arginine methylation, to symmetrical-dimethyl arginine
GO:0019919	peptidyl-arginine methylation, to asymmetrical-dimethyl arginine
GO:0030466	chromatin silencing at silent mating-type cassette
GO:0030488	tRNA methylation
GO:0030561	RNA 2'-O-ribose methylation guide activity
GO:0030562	rRNA 2'-O-ribose methylation guide activity
GO:0030563	snRNA 2'-O-ribose methylation guide activity
GO:0030564	tRNA 2'-O-ribose methylation guide activity
GO:0030702	chromatin silencing at centromere
GO:0031048	chromatin silencing by small RNA
GO:0031055	chromatin remodeling at centromere
GO:0031056	regulation of histone modification
GO:0031057	negative regulation of histone modification
GO:0031058	positive regulation of histone modification
GO:0031059	histone deacetylation at centromere
GO:0031060	regulation of histone methylation
GO:0031061	negative regulation of histone methylation
GO:0031062	positive regulation of histone methylation
GO:0031063	regulation of histone deacetylation
GO:0031064	negative regulation of histone deacetylation
GO:0031065	positive regulation of histone deacetylation
GO:0031066	regulation of histone deacetylation at centromere
GO:0031067	negative regulation of histone deacetylation at centromere
GO:0031068	positive regulation of histone deacetylation at centromere
GO:0031078	histone deacetylase activity (H3-K14 specific)
GO:0031151	histone methyltransferase activity (H3-K79 specific)
GO:0031167	rRNA methylation
GO:0031445	regulation of heterochromatin assembly
GO:0031452	negative regulation of heterochromatin assembly
GO:0031453	positive regulation of heterochromatin assembly
GO:0031454	regulation of extent of heterochromatin assembly
GO:0031490	chromatin DNA binding
GO:0031493	nucleosomal histone binding
GO:0031497	chromatin assembly
GO:0031498	chromatin disassembly
GO:0031507	heterochromatin assembly
GO:0031508	pericentric heterochromatin assembly
GO:0031509	telomeric heterochromatin assembly
GO:0031935	regulation of chromatin silencing
GO:0031936	negative regulation of chromatin silencing
GO:0031937	positive regulation of chromatin silencing

GO:0031938	regulation of chromatin silencing at telomere
GO:0031939	negative regulation of chromatin silencing at telomere
GO:0031940	positive regulation of chromatin silencing at telomere
GO:0032041	NAD-dependent histone deacetylase activity (H3-K14 specific)
GO:0032121	meiotic attachment of telomeric heterochromatin to spindle pole body
GO:0032129	histone deacetylase activity (H3-K9 specific)
GO:0032259	Methylation
GO:0032452	histone demethylase activity
GO:0032453	histone demethylase activity (H3-K4 specific)
GO:0032454	histone demethylase activity (H3-K9 specific)
GO:0032775	DNA methylation on adenine
GO:0032776	DNA methylation on cytosine
GO:0032931	histone acetyltransferase activity (H3-K56 specific)
GO:0033127	regulation of histone phosphorylation
GO:0033128	negative regulation of histone phosphorylation
GO:0033129	positive regulation of histone phosphorylation
GO:0033169	histone H3-K9 demethylation
GO:0033182	regulation of histone ubiquitination
GO:0033183	negative regulation of histone ubiquitination
GO:0033184	positive regulation of histone ubiquitination
GO:0033522	histone H2A ubiquitination
GO:0033523	histone H2B ubiquitination
GO:0033553	rDNA heterochromatin
GO:0033696	negative regulation of extent of heterochromatin assembly
GO:0033697	positive regulation of extent of heterochromatin assembly
GO:0033746	histone demethylase activity (H3-R2 specific)
GO:0033749	histone demethylase activity (H4-R3 specific)
GO:0034401	chromatin organization involved in regulation of transcription
GO:0034647	histone demethylase activity (H3-trimethyl-K4 specific)
GO:0034648	histone demethylase activity (H3-dimethyl-K4 specific)
GO:0034649	histone demethylase activity (H3-monomethyl-K4 specific)
GO:0034720	histone H3-K4 demethylation
GO:0034721	histone H3-K4 demethylation, trimethyl-H3-K4-specific
GO:0034729	histone H3-K79 methylation
GO:0034739	histone deacetylase activity (H4-K16 specific)
GO:0034770	histone H4-K20 methylation
GO:0034771	histone H4-K20 monomethylation
GO:0034772	histone H4-K20 dimethylation
GO:0034773	histone H4-K20 trimethylation
GO:0034968	histone lysine methylation
GO:0034969	histone arginine methylation
GO:0034970	histone H3-R2 methylation
GO:0034971	histone H3-R17 methylation
GO:0034972	histone H3-R26 methylation
GO:0035033	histone deacetylase regulator activity
GO:0035034	histone acetyltransferase regulator activity

GO:0035035	histone acetyltransferase binding
GO:0035041	sperm chromatin decondensation
GO:0035064	methylated histone binding
GO:0035065	regulation of histone acetylation
GO:0035066	positive regulation of histone acetylation
GO:0035067	negative regulation of histone acetylation
GO:0035092	sperm chromatin condensation
GO:0035173	histone kinase activity
GO:0035174	histone serine kinase activity
GO:0035175	histone kinase activity (H3-S10 specific)
GO:0035184	histone threonine kinase activity
GO:0035245	peptidyl-arginine C-methylation
GO:0035246	peptidyl-arginine N-methylation
GO:0035247	peptidyl-arginine omega-N-methylation
GO:0035327	transcriptionally active chromatin
GO:0035328	transcriptionally silent chromatin
GO:0035389	establishment of chromatin silencing at silent mating-type cassette
GO:0035390	establishment of chromatin silencing at telomere
GO:0035391	maintenance of chromatin silencing at silent mating-type cassette
GO:0035392	maintenance of chromatin silencing at telomere
GO:0035400	histone tyrosine kinase activity
GO:0035401	histone kinase activity (H3-Y41 specific)
GO:0035402	histone kinase activity (H3-T11 specific)
GO:0035403	histone kinase activity (H3-T6 specific)
GO:0035404	histone-serine phosphorylation
GO:0035405	histone-threonine phosphorylation
GO:0035406	histone-tyrosine phosphorylation
GO:0035407	histone H3-T11 phosphorylation
GO:0035408	histone H3-T6 phosphorylation
GO:0035409	histone H3-Y41 phosphorylation
GO:0035511	oxidative DNA demethylation
GO:0035512	hydrolytic DNA demethylation
GO:0035513	oxidative RNA demethylation
GO:0035518	histone H2A monoubiquitination
GO:0035521	monoubiquitinated histone deubiquitination
GO:0035522	monoubiquitinated histone H2A deubiquitination
GO:0035552	oxidative single-stranded DNA demethylation
GO:0035553	oxidative single-stranded RNA demethylation
GO:0035561	regulation of chromatin binding
GO:0035562	negative regulation of chromatin binding
GO:0035563	positive regulation of chromatin binding
GO:0035568	N-terminal peptidyl-proline methylation
GO:0035570	N-terminal peptidyl-serine methylation
GO:0035571	N-terminal peptidyl-serine monomethylation
GO:0035572	N-terminal peptidyl-serine dimethylation

GO:0035573 N-terminal peptidyl-serine trimethylation
GO:0035574 histone H4-K20 demethylation
GO:0035575 histone demethylase activity (H4-K20 specific)
GO:0035616 histone H2B conserved C-terminal lysine deubiquitination
GO:0035642 histone methyltransferase activity (H3-R17 specific)
GO:0035978 histone H2A-S139 phosphorylation
GO:0035979 histone kinase activity (H2A-S139 specific)
GO:0035985 senescence-associated heterochromatin focus
GO:0035986 senescence-associated heterochromatin focus assembly
GO:0036123 histone H3-K9 dimethylation
GO:0036124 histone H3-K9 trimethylation
GO:0036205 histone catabolic process
GO:0036206 regulation of histone gene expression
GO:0036207 positive regulation of histone gene expression
GO:0036208 negative regulation of histone gene expression
GO:0036261 7-methylguanosine cap hypermethylation
GO:0036265 RNA (guanine-N7)-methylation
GO:0036351 histone H2A-K13 ubiquitination
GO:0036352 histone H2A-K15 ubiquitination
GO:0036353 histone H2A-K119 monoubiquitination
GO:0036408 histone acetyltransferase activity (H3-K14 specific)
GO:0036413 histone H3-R26 citrullination
GO:0036414 histone citrullination
GO:0036451 cap mRNA methylation
GO:0039525 modulation by virus of host chromatin organization
GO:0039699 viral mRNA cap methylation
GO:0040029 regulation of gene expression, epigenetic
GO:0040030 regulation of molecular function, epigenetic
GO:0042037 peptidyl-histidine methylation, to form pros-methylhistidine
GO:0042038 peptidyl-histidine methylation, to form tele-methylhistidine
GO:0042054 histone methyltransferase activity
GO:0042393 histone binding
GO:0042799 histone methyltransferase activity (H4-K20 specific)
GO:0042800 histone methyltransferase activity (H3-K4 specific)
GO:0042826 histone deacetylase binding
GO:0043035 chromatin insulator sequence binding
GO:0043044 ATP-dependent chromatin remodeling
GO:0043045 DNA methylation involved in embryo development
GO:0043046 DNA methylation involved in gamete generation
GO:0043156 chromatin remodeling in response to cation stress
GO:0043189 H4/H2A histone acetyltransferase complex
GO:0043414 macromolecule methylation
GO:0043486 histone exchange
GO:0043966 histone H3 acetylation
GO:0043967 histone H4 acetylation
GO:0043968 histone H2A acetylation

GO:0043969	histone H2B acetylation
GO:0043970	histone H3-K9 acetylation
GO:0043971	histone H3-K18 acetylation
GO:0043972	histone H3-K23 acetylation
GO:0043973	histone H3-K4 acetylation
GO:0043974	histone H3-K27 acetylation
GO:0043975	histone H3-K36 acetylation
GO:0043976	histone H3-K79 acetylation
GO:0043977	histone H2A-K5 acetylation
GO:0043978	histone H2A-K9 acetylation
GO:0043979	histone H2B-K5 acetylation
GO:0043980	histone H2B-K12 acetylation
GO:0043981	histone H4-K5 acetylation
GO:0043982	histone H4-K8 acetylation
GO:0043983	histone H4-K12 acetylation
GO:0043984	histone H4-K16 acetylation
GO:0043985	histone H4-R3 methylation
GO:0043987	histone H3-S10 phosphorylation
GO:0043988	histone H3-S28 phosphorylation
GO:0043989	histone H4-S1 phosphorylation
GO:0043990	histone H2A-S1 phosphorylation
GO:0043991	histone H2B-S14 phosphorylation
GO:0043992	histone acetyltransferase activity (H3-K9 specific)
GO:0043993	histone acetyltransferase activity (H3-K18 specific)
GO:0043994	histone acetyltransferase activity (H3-K23 specific)
GO:0043995	histone acetyltransferase activity (H4-K5 specific)
GO:0043996	histone acetyltransferase activity (H4-K8 specific)
GO:0043997	histone acetyltransferase activity (H4-K12 specific)
GO:0043998	H2A histone acetyltransferase activity
GO:0043999	histone acetyltransferase activity (H2A-K5 specific)
GO:0044012	histone acetyltransferase activity (H2A-K9 specific)
GO:0044013	H2B histone acetyltransferase activity
GO:0044014	histone acetyltransferase activity (H2B-K5 specific)
GO:0044015	histone acetyltransferase activity (H2B-K12 specific)
GO:0044016	histone acetyltransferase activity (H3-K4 specific)
GO:0044017	histone acetyltransferase activity (H3-K27 specific)
GO:0044018	histone acetyltransferase activity (H3-K36 specific)
GO:0044019	histone acetyltransferase activity (H3-K72 specific)
GO:0044020	histone methyltransferase activity (H4-R3 specific)
GO:0044022	histone kinase activity (H3-S28 specific)
GO:0044023	histone kinase activity (H4-S1 specific)
GO:0044024	histone kinase activity (H2A-S1 specific)
GO:0044025	histone kinase activity (H2B-S14 specific)
GO:0044026	DNA hypermethylation
GO:0044027	hypermethylation of CpG island

GO:0044028 DNA hypomethylation
GO:0044029 hypomethylation of CpG island
GO:0044030 regulation of DNA methylation
GO:0044154 histone H3-K14 acetylation
GO:0044382 CLRC ubiquitin ligase complex localization to heterochromatin
GO:0044648 histone H3-K4 dimethylation
GO:0044725 chromatin reprogramming in the zygote
GO:0044726 protection of DNA demethylation of female pronucleus
GO:0044727 DNA demethylation of male pronucleus
GO:0044728 DNA methylation or demethylation
GO:0045129 NAD-independent histone deacetylase activity
GO:0045798 negative regulation of chromatin assembly or disassembly
GO:0045799 positive regulation of chromatin assembly or disassembly
GO:0045814 negative regulation of gene expression, epigenetic
GO:0045815 positive regulation of gene expression, epigenetic
GO:0045857 negative regulation of molecular function, epigenetic
GO:0045858 positive regulation of molecular function, epigenetic
GO:0046811 histone deacetylase inhibitor activity
GO:0046895 N-terminal peptidyl-isoleucine methylation
GO:0046896 N-terminal peptidyl-leucine methylation
GO:0046897 N-terminal peptidyl-tyrosine methylation
GO:0046969 NAD-dependent histone deacetylase activity (H3-K9 specific)
GO:0046970 NAD-dependent histone deacetylase activity (H4-K16 specific)
GO:0046972 histone acetyltransferase activity (H4-K16 specific)
GO:0046974 histone methyltransferase activity (H3-K9 specific)
GO:0046975 histone methyltransferase activity (H3-K36 specific)
GO:0046976 histone methyltransferase activity (H3-K27 specific)
GO:0048096 chromatin-mediated maintenance of transcription
GO:0050838 peptidyl-5-hydroxy-L-lysine trimethylation
GO:0051567 histone H3-K9 methylation
GO:0051568 histone H3-K4 methylation
GO:0051569 regulation of histone H3-K4 methylation
GO:0051570 regulation of histone H3-K9 methylation
GO:0051571 positive regulation of histone H3-K4 methylation
GO:0051572 negative regulation of histone H3-K4 methylation
GO:0051573 negative regulation of histone H3-K9 methylation
GO:0051574 positive regulation of histone H3-K9 methylation
GO:0051864 histone demethylase activity (H3-K36 specific)
GO:0060820 inactivation of X chromosome by heterochromatin assembly
GO:0060821 inactivation of X chromosome by DNA methylation
GO:0060906 negative regulation of chromatin silencing by small RNA
GO:0061085 regulation of histone H3-K27 methylation
GO:0061086 negative regulation of histone H3-K27 methylation
GO:0061087 positive regulation of histone H3-K27 methylation
GO:0061186 negative regulation of chromatin silencing at silent mating-type cassette
GO:0061187 regulation of chromatin silencing at rDNA

GO:0061188	negative regulation of chromatin silencing at rDNA
GO:0061628	H3K27me3 modified histone binding
GO:0061638	CENP-A containing chromatin
GO:0061641	CENP-A containing chromatin organization
GO:0061644	protein localization to CENP-A containing chromatin
GO:0061647	histone H3-K9 modification
GO:0061649	ubiquitin modification-dependent histone binding
GO:0061715	miRNA 2'-O-methylation
GO:0061866	negative regulation of histone H3-S10 phosphorylation
GO:0061922	histone propionyltransferase activity
GO:0062060	NuA4 histone acetyltransferase complex binding
GO:0062072	H3K9me3 modified histone binding
GO:0070076	histone lysine demethylation
GO:0070077	histone arginine demethylation
GO:0070078	histone H3-R2 demethylation
GO:0070079	histone H4-R3 demethylation
GO:0070475	rRNA base methylation
GO:0070476	rRNA (guanine-N7)-methylation
GO:0070510	regulation of histone H4-K20 methylation
GO:0070511	negative regulation of histone H4-K20 methylation
GO:0070512	positive regulation of histone H4-K20 methylation
GO:0070535	histone H2A K63-linked ubiquitination
GO:0070537	histone H2A K63-linked deubiquitination
GO:0070544	histone H3-K36 demethylation
GO:0070577	lysine-acetylated histone binding
GO:0070611	histone methyltransferase activity (H3-R2 specific)
GO:0070612	histone methyltransferase activity (H2A-R3 specific)
GO:0070734	histone H3-K27 methylation
GO:0070775	H3 histone acetyltransferase complex
GO:0070827	chromatin maintenance
GO:0070828	heterochromatin organization
GO:0070829	heterochromatin maintenance
GO:0070868	heterochromatin organization involved in chromatin silencing
GO:0070869	heterochromatin assembly involved in chromatin silencing
GO:0070870	heterochromatin maintenance involved in chromatin silencing
GO:0070901	mitochondrial tRNA methylation
GO:0070919	production of siRNA involved in chromatin silencing by small RNA
GO:0070921	regulation of production of siRNA involved in chromatin silencing by small RNA
GO:0070923	siRNA loading onto RISC involved in chromatin silencing by small RNA
GO:0070924	heterochromatin assembly involved in chromatin silencing by small RNA
GO:0070932	histone H3 deacetylation
GO:0070933	histone H4 deacetylation
GO:0070988	Demethylation
GO:0070989	oxidative demethylation
GO:0071044	histone mRNA catabolic process

GO:0071110	histone biotinylation
GO:0071168	protein localization to chromatin
GO:0071169	establishment of protein localization to chromatin
GO:0071207	histone pre-mRNA stem-loop binding
GO:0071208	histone pre-mRNA DCP binding
GO:0071440	regulation of histone H3-K14 acetylation
GO:0071441	negative regulation of histone H3-K14 acetylation
GO:0071442	positive regulation of histone H3-K14 acetylation
GO:0071557	histone H3-K27 demethylation
GO:0071558	histone demethylase activity (H3-K27 specific)
GO:0071572	histone H3-K56 deacetylation
GO:0071891	N-terminal peptidyl-proline dimethylation involved in translation
GO:0071894	histone H2B conserved C-terminal lysine ubiquitination
GO:0072354	histone kinase activity (H3-T3 specific)
GO:0072355	histone H3-T3 phosphorylation
GO:0072370	histone H2A-S121 phosphorylation
GO:0072371	histone kinase activity (H2A-S121 specific)
GO:0080009	mRNA methylation
GO:0080111	DNA demethylation
GO:0080182	histone H3-K4 trimethylation
GO:0080188	RNA-directed DNA methylation
GO:0090052	regulation of chromatin silencing at centromere
GO:0090053	positive regulation of chromatin silencing at centromere
GO:0090054	regulation of chromatin silencing at silent mating-type cassette
GO:0090055	positive regulation of chromatin silencing at silent mating-type cassette
GO:0090115	C-5 methylation on cytosine involved in chromatin silencing
GO:0090116	C-5 methylation of cytosine
GO:0090124	N-4 methylation of cytosine
GO:0090223	chromatin-templated microtubule nucleation
GO:0090239	regulation of histone H4 acetylation
GO:0090240	positive regulation of histone H4 acetylation
GO:0090241	negative regulation of histone H4 acetylation
GO:0090308	regulation of methylation-dependent chromatin silencing
GO:0090309	positive regulation of methylation-dependent chromatin silencing
GO:0090310	negative regulation of methylation-dependent chromatin silencing
GO:0097043	histone H3-K56 acetylation
GO:0097044	histone H3-K56 acetylation in response to DNA damage
GO:0097198	histone H3-K36 trimethylation
GO:0097309	cap1 mRNA methylation
GO:0097310	cap2 mRNA methylation
GO:0097355	protein localization to heterochromatin
GO:0097372	NAD-dependent histone deacetylase activity (H3-K18 specific)
GO:0097424	nucleolus-associated heterochromatin
GO:0097549	chromatin organization involved in negative regulation of transcription
GO:0097676	histone H3-K36 dimethylation
GO:0097692	histone H3-K4 monomethylation

GO:0097725	histone H3-K79 dimethylation
GO:0098532	histone H3-K27 trimethylation
GO:0098578	condensed chromatin of inactivated sex chromosome
GO:0099077	histone-dependent DNA binding
GO:0099114	chromatin silencing at subtelomere
GO:0106004	tRNA (guanine-N7)-methylation
GO:0106005	RNA 5'-cap (guanine-N7)-methylation
GO:0106077	histone succinylation
GO:0106078	histone succinyltransferase activity
GO:0106153	phosphorylated histone binding
GO:0110002	regulation of tRNA methylation
GO:0110003	regulation of tRNA C5-cytosine methylation
GO:0110004	positive regulation of tRNA methylation
GO:0110005	positive regulation of tRNA C5-cytosine methylation
GO:0120049	snRNA (adenine-N6)-methylation
GO:0120186	negative regulation of protein localization to chromatin
GO:0120187	positive regulation of protein localization to chromatin
GO:0140034	methylation-dependent protein binding
GO:0140068	histone crotonyltransferase activity
GO:0140069	histone butyryltransferase activity
GO:1900049	regulation of histone exchange
GO:1900050	negative regulation of histone exchange
GO:1900051	positive regulation of histone exchange
GO:1900109	regulation of histone H3-K9 dimethylation
GO:1900110	negative regulation of histone H3-K9 dimethylation
GO:1900111	positive regulation of histone H3-K9 dimethylation
GO:1900112	regulation of histone H3-K9 trimethylation
GO:1900113	negative regulation of histone H3-K9 trimethylation
GO:1900114	positive regulation of histone H3-K9 trimethylation
GO:1901314	regulation of histone H2A K63-linked ubiquitination
GO:1901315	negative regulation of histone H2A K63-linked ubiquitination
GO:1901316	positive regulation of histone H2A K63-linked ubiquitination
GO:1901535	regulation of DNA demethylation
GO:1901536	negative regulation of DNA demethylation
GO:1901537	positive regulation of DNA demethylation
GO:1901538	changes to DNA methylation involved in embryo development
GO:1901674	regulation of histone H3-K27 acetylation
GO:1901675	negative regulation of histone H3-K27 acetylation
GO:1901676	positive regulation of histone H3-K27 acetylation
GO:1901725	regulation of histone deacetylase activity
GO:1901726	negative regulation of histone deacetylase activity
GO:1901727	positive regulation of histone deacetylase activity
GO:1902028	regulation of histone H3-K18 acetylation
GO:1902029	positive regulation of histone H3-K18 acetylation
GO:1902030	negative regulation of histone H3-K18 acetylation

GO:1902275	regulation of chromatin organization
GO:1902360	conversion of ds siRNA to ss siRNA involved in chromatin silencing by small RNA
GO:1902368	heterochromatin maintenance involved in chromatin silencing at centromere outer repeat region
GO:1902464	regulation of histone H3-K27 trimethylation
GO:1902465	negative regulation of histone H3-K27 trimethylation
GO:1902466	positive regulation of histone H3-K27 trimethylation
GO:1902562	H4 histone acetyltransferase complex
GO:1902649	regulation of histone H2A-H2B dimer displacement
GO:1902650	negative regulation of histone H2A-H2B dimer displacement
GO:1902651	positive regulation of histone H2A-H2B dimer displacement
GO:1902794	heterochromatin island assembly
GO:1902795	heterochromatin domain assembly
GO:1902801	regulation of heterochromatin island assembly
GO:1902802	regulation of heterochromatin domain assembly
GO:1903212	protein localization to mating-type region heterochromatin
GO:1903213	protein localization to subtelomeric heterochromatin
GO:1903584	regulation of histone deubiquitination
GO:1903585	negative regulation of histone deubiquitination
GO:1903586	positive regulation of histone deubiquitination
GO:1903756	regulation of transcription from RNA polymerase II promoter by histone modification
GO:1903757	positive regulation of transcription from RNA polymerase II promoter by histone modification
GO:1903758	negative regulation of transcription from RNA polymerase II promoter by histone modification
GO:1904173	regulation of histone demethylase activity (H3-K4 specific)
GO:1904174	negative regulation of histone demethylase activity (H3-K4 specific)
GO:1904175	positive regulation of histone demethylase activity (H3-K4 specific)
GO:1904497	heterochromatin assembly involved in chromatin silencing at centromere outer repeat region
GO:1904499	regulation of chromatin-mediated maintenance of transcription
GO:1904500	negative regulation of chromatin-mediated maintenance of transcription
GO:1904501	positive regulation of chromatin-mediated maintenance of transcription
GO:1904793	regulation of euchromatin binding
GO:1904794	negative regulation of euchromatin binding
GO:1904795	positive regulation of euchromatin binding
GO:1905268	negative regulation of chromatin organization
GO:1905269	positive regulation of chromatin organization
GO:1905435	regulation of histone H3-K4 trimethylation
GO:1905436	negative regulation of histone H3-K4 trimethylation
GO:1905437	positive regulation of histone H3-K4 trimethylation
GO:1905471	regulation of histone H3-K79 dimethylation
GO:1905472	negative regulation of histone H3-K79 dimethylation
GO:1905473	positive regulation of histone H3-K79 dimethylation
GO:1905547	regulation of telomeric heterochromatin assembly
GO:1905548	negative regulation of telomeric heterochromatin assembly
GO:1905549	positive regulation of telomeric heterochromatin assembly
GO:1905632	protein localization to euchromatin

GO:1905633	establishment of protein localization to euchromatin
GO:1905634	regulation of protein localization to chromatin
GO:1905642	negative regulation of DNA methylation
GO:1905643	positive regulation of DNA methylation
GO:1905886	chromatin remodeling involved in meiosis I
GO:1990141	chromatin silencing at centromere outer repeat region
GO:1990152	protein localization to telomeric heterochromatin
GO:1990153	maintenance of protein localization to heterochromatin
GO:1990162	histone deacetylase activity (H3-K4 specific)
GO:1990164	histone H2A phosphorylation
GO:1990188	euchromatin binding
GO:1990226	histone methyltransferase binding
GO:1990244	histone kinase activity (H2A-T120 specific)
GO:1990245	histone H2A-T120 phosphorylation
GO:1990258	histone glutamine methylation
GO:1990259	histone-glutamine methyltransferase activity
GO:1990280	RNA localization to chromatin
GO:1990437	snRNA 2'-O-methylation
GO:1990438	U6 2'-O-snRNA methylation
GO:1990577	C-terminal protein demethylation
GO:1990596	histone H3-K4 deacetylation
GO:1990619	histone H3-K9 deacetylation
GO:1990678	histone H4-K16 deacetylation
GO:1990679	histone H4-K12 deacetylation
GO:1990700	nucleolar chromatin organization
GO:1990744	primary miRNA methylation
GO:1990841	promoter-specific chromatin binding
GO:1990853	histone H2A SQE motif phosphorylation
GO:1990889	H4K20me3 modified histone binding
GO:1990983	tRNA demethylation
GO:2000281	regulation of histone H3-T3 phosphorylation
GO:2000615	regulation of histone H3-K9 acetylation
GO:2000616	negative regulation of histone H3-K9 acetylation
GO:2000617	positive regulation of histone H3-K9 acetylation
GO:2000618	regulation of histone H4-K16 acetylation
GO:2000619	negative regulation of histone H4-K16 acetylation
GO:2000620	positive regulation of histone H4-K16 acetylation
GO:2000749	positive regulation of chromatin silencing at rDNA
GO:2000751	histone H3-T3 phosphorylation involved in chromosome passenger complex localization to kinetochore
GO:2000775	histone H3-S10 phosphorylation involved in chromosome condensation
GO:2000776	histone H4 acetylation involved in response to DNA damage stimulus
GO:2000817	regulation of histone H3-T3 phosphorylation involved in chromosome passenger complex localization to kinetochore
GO:2000873	regulation of histone H4 acetylation involved in response to DNA damage stimulus

GO:2001160 regulation of histone H3-K79 methylation
GO:2001161 negative regulation of histone H3-K79 methylation
GO:2001162 positive regulation of histone H3-K79 methylation
GO:2001166 regulation of histone H2B ubiquitination
GO:2001167 negative regulation of histone H2B ubiquitination
GO:2001168 positive regulation of histone H2B ubiquitination
GO:2001173 regulation of histone H2B conserved C-terminal lysine ubiquitination
GO:2001174 negative regulation of histone H2B conserved C-terminal lysine ubiquitination
GO:2001175 positive regulation of histone H2B conserved C-terminal lysine ubiquitination
GO:2001253 regulation of histone H3-K36 trimethylation
GO:2001254 negative regulation of histone H3-K36 trimethylation
GO:2001255 positive regulation of histone H3-K36 trimethylation

Supplementary Table 3. Human and murine genes related to epigenetics mechanisms

***Homo sapiens* genes**
ABRAXAS1, AC006064.6, AC007998.2, AC068896.3, AC069288.1, AC099811.2, AC106886.6, AC120114.4, ACTB, ACTL6A, ACTL6B, ACTN4, ACTR5, ACTR6, ACTR8, ACTRT1, ADNP, AEBP2, AHCY, AICDA, AIRE, AJUBA, AKAP8, AKAP8L, AL031708.1, AL031777.2, AL133500.1, ALKBH1, ALKBH2, ALKBH3, ALKBH4, ALKBH5, ALKBH8, AMT, ANKRA2, ANKRD1, ANKRD17, ANKRD2, ANP32A, ANP32B, ANP32E, ANTKMT, AP001267.5, APBB1, APEX1, APOBEC1, APOBEC2, APOBEC3A, APOBEC3B, APOBEC3C, APOBEC3D, APOBEC3F, APOBEC3G, APOBEC3H, APTX, AR, ARID1A, ARID1B, ARID2, ARID3A, ARID3C, ARID4A, ARID4B, ARMT1, ARRB1, ARX, AS3MT, ASCL1, ASF1A, ASF1B, ASH1L, ASH2L, ASIP, ASMT, ASMTL, ASXL1, ASXL2, ASXL3, ASZ1, ATAD2, ATAD2B, ATF2, ATF5, ATF7IP, ATG5, ATM, ATOH1, ATPSCKMT, ATRX, ATXN3, ATXN7, ATXN7L3, AURKA, AURKB, AURKC, AUTS2, BABAM1, BABAM2, BAG6, BAHCC1, BAHD1, BANP, BAP1, BARX2, BAZ1A, BAZ1B, BAZ2A, BAZ2B, BCAS3, BCDIN3D, BCL6, BCOR, BCORL1, BEND3, BEND6, BEX4, BHLHE41, BHMT, BHMT2, BMI1, BMT2, BORCS8-MEF2B, BPTF, BRCA1, BRCA2, BRCC3, BRD1, BRD2, BRD3, BRD4, BRD7, BRD8, BRD9, BRDT, BRIP1, BRMS1, BRMS1L, BRPF1, BRPF3, BRWD1, BTAF1, BTG2, BUD23, C17orf49, C6orf89, CABIN1, CALCOCO1, CAMK2D, CAMKMT, CAMTA2, CARM1, CARNMT1, CBLL1, CBX1, CBX2, CBX3, CBX4, CBX5, CBX6, CBX7, CBX8, CCNA2, CCNB1, CCND1, CCNT1, CCNT2, CD1D, CD3EAP, CDAN1, CDC20, CDC45, CDC73, CDCA5, CDK1, CDK2, CDK5, CDK9, CDKN2A, CDT1, CDY1, CDY1B, CDY2A, CDY2B, CDYL, CDYL2, CEBPA, CEBPB, CECR2, CENPA, CENPB, CENPF, CENPS, CENPV, CGAS, CHAF1A, CHAF1B, CHD1, CHD1L, CHD2, CHD3, CHD4, CHD5, CHD6, CHD7, CHD8, CHD9, CHEK1, CHMP7, CHRAC1, CHTOP, CIC, CIR1, CITED1, CITED2, CKS1B, CKS2, CLOCK, CMTR1, CMTR2, COMMD3-BMI1, COMT, COMTD1, COPRS, COQ3, COQ5, CPA4, CRAMP1, CREB1, CREB3L1, CREBBP, CREBZF, CRTC2, CRY1, CSKMT, CSNK2B, CTBP1, CTBP2, CTCF, CTCFL, CTNNB1, CTR9, CTSL, CUL4B, CXXC1, CYP1A1, CYP1A2, CYP2C8, CYP2C9, CYP2D6, CYP3A4, CYP3A43, CYP3A5, CYP3A7, CYP51A1, DACT1, DAPK3, DAXX, DCAF1, DCP2, DDB1, DDB2, DDX1, DDX11, DDX20, DDX21, DDX4, DDX5, DEK, DHX30, DHX36, DHX9, DIMT1, DLD, DLST, DLX1, DLX2, DLX3, DMAP1, DMRT1, DMRTC2, DNAJC2, DNMT1, DNMT3A, DNMT3B, DNMT3L, DOT1L, DPF1, DPF2, DPF3, DPH5, DPPA2, DPPA3, DPPA4, DPY30, DR1, DRD1, DTX3L, DYDC1, DYDC2, E2F4, EBF2, ECD, ECE2, EED, EEF1AKMT1, EEF1AKMT2, EEF1AKMT3, EEF1AKMT4, EEF1AKMT4-ECE2, EEF1AKNMT, EEF2KMT, EGFR, EGR1, EGR2, EHMT1, EHMT2, EID1, ELK1, ELK4, ELOF1, EMG1, EMSY, ENY2, EOMES, EP300, EP400, EPAS1, EPC1, EPC2, EPOP, ERCC1, ERCC3, ERCC4, ERCC6, ERG, ERI1, ESCO2, ESR1, ETF1, ETFBKMT, ETS1, EXO1, EXOSC10, EXOSC4, EYA1, EYA2, EYA3, EYA4, EZH1, EZH2, EZHIP, FAAP24, FABP1, FAM156A, FAM156B, FAM172A, FAM50A, FAM50B, FAM86B1, FAM86B2, FAM86C1, FAM98A, FAM98B, FANCM, FBH1, FBL, FBLL1, FBXO11, FDXACB1, FEZF2, FKBP6, FLCN, FLI1, FMR1, FOS, FOSL2, FOXA1, FOXA2, FOXA3, FOXC1, FOXC2, FOXN4, FOXO1, FOXO3, FOXO4, FOXP3, FP565260.4, FTO, FTSJ1, FTSJ3, FUS, GABPA, GADD45A, GAMT, GATA1, GATA2, GATA3, GATA6, GATAD1, GATAD2A, GATAD2B, GCG, GCM1, GFI1, GFI1B, GLI1, GLI2, GLI3, GLMN, GLYR1, GMNC, GMNN, GNAS, GNMT, GPER1, GPX1, GPX4, GRHL1, GRHL2, GRHL3, GRWD1, GSPT1, GSTO1, GTF2B, GTF2F1, GTF2H1, GTF3C4, GTPBP3, H1-0, H1-1, H1-10, H1-2, H1-3, H1-4, H1-5, H1-6, H1-7, H1-8, H2AB1, H2AB2, H2AB3, H2AC1, H2AC11, H2AC12, H2AC13, H2AC14, H2AC15, H2AC16, H2AC17, H2AC18, H2AC19, H2AC20, H2AC21, H2AC4, H2AC6, H2AC7, H2AC8, H2AJ, H2AP, H2AW, H2AX, H2AZ1, H2AZ2, H2BC1, H3-3A, H3-3B, H3C1, H3C10, H3C11, H3C12, H3C13, H3C14, H3C15, H3C2, H3C3, H3C4, H3C6, H3C7, H3C8, H4-16, H4C1, H4C11, H4C12, H4C13, H4C14, H4C15, H4C2, H4C3, H4C4, H4C5, H4C6, H4C8, H4C9, HASPIN, HAT1, HCFC1, HCFC2, HDAC1, HDAC10, HDAC11, HDAC2, HDAC3, HDAC4, HDAC5, HDAC6, HDAC7, HDAC8, HDAC9, HELLS, HEMK1, HENMT1, HES1, HES5, HESX1, HEY2, HIC1, HIF1A, HINFP, HIPK4, HIRA, HIRIP3, HJURP, HLCS, HLTF, HMBOX1, HMG20A, HMG20B, HMGA1, HMGA2, HMGB1, HMGB2, HMGB3, HMGB4, HMGN3, HMGN5, HNF1A, HNF4A, HNMT, HNRNPC, HNRNPD, HNRNPU, HOXA10, HOXC13, HOXD10, HOXD13, HP1BP3, HPF1, HR, HSD17B10, HSF1, HSF4, HSP90AA1, HSP90AB1, HSPA1A, HSPA1B, HUWE1, ICMT, IFI16, IFT74, IGF2, IKZF1, IKZF3, IL1B, INCENP, ING1, ING2, ING3, ING4, ING5, INMT, INO80, INO80B, INO80B-WBP1, INO80C, INO80E, INSM1, IPO7, IPO9, IRF4, ISL1, IWS1, JADE1, JADE2, JADE3, JAK2, JARID2, JDP2, JMJD1C, JMJD6, JMJD7, JUN, KANSL1, KANSL1L, KANSL2, KANSL3, KAT14, KAT2A, KAT2B, KAT5, KAT6A, KAT6B, KAT7, KAT8, KCTD21, KDM1A, KDM1B, KDM2A, KDM2B, KDM3A, KDM3B, KDM4A, KDM4B, KDM4C, KDM4D, KDM4E, KDM4F, KDM5A, KDM5B, KDM5C, KDM5D, KDM6A, KDM6B, KDM7A, KDM8, KLF14, KLF2, KLF4, KLHDC3, KMT2A, KMT2B, KMT2C, KMT2D, KMT2E, KMT5A, KMT5B, KMT5C, KPNA2, KPNA7, L3MBTL1, L3MBTL2, L3MBTL3, L3MBTL4, LCMT1, LCMT2, LDB1, LEF1, LEMD2, LEO1, LHX2, LIF, LMNA, LOXL2, LRRK2, LRTOMT, LRWD1, LSM1, LSM10, LSM11, M1AP, MACROH2A1, MACROH2A2, MAEL,

MAGEA1, MAGEA2, MAGEA2B, MAP1S, MAP3K12, MAP3K7, MAPK15, MAPK3, MAPK8, MAPT, MAT1A, MAT2A, MAT2B, MBD1, MBD2, MBD3, MBD3L1, MBD3L2, MBD3L2B, MBD3L3, MBD3L4, MBD3L5, MBD5, MBD6, MBIP, MBTD1, MCM2, MCM3AP, MCM8, MCM9, MCMBP, MCRS1, MEAF6, MECOM, MECP2, MED1, MED12, MED24, MED25, MEF2A, MEF2B, MEF2C, MEF2D, MEIOB, MEIS1, MEIS3, MEN1, MEOX1, MEPCE, METTL1, METTL11B, METTL14, METTL15, METTL16, METTL17, METTL18, METTL21A, METTL21C, METTL22, METTL23, METTL24, METTL25, METTL2A, METTL2B, METTL3, METTL4, METTL5, METTL6, METTL7A, METTL7B, METTL8, MGMT, MIER1, MIER2, MIER3, MIR182, MIR29A, MIR29B1, MIR29B2, MIR29C, MIS18A, MITF, MKI67, MLH1, MLH3, MLLT10, MLLT3, MLLT6, MMACHC, MNT, MORC1, MORC2, MORF4L1, MORF4L2, MOS, MOV10L1, MPHOSPH8, MPO, MRGBP, MRM1, MRM2, MRM3, MRNIP, MSGN1, MSH2, MSH6, MSL1, MSL2, MSL3, MT3, MTA1, MTA2, MTA3, MTERF4, MTF1, MTF2, MTHFR, MTO1, MTPAP, MTR, MTRR, MUC1, MYB, MYBBP1A, MYC, MYO1C, MYOCD, MYOD1, MYOG, MYSM1, N6AMT1, NAA40, NAA50, NAA60, NACC1, NACC2, NAP1L2, NASP, NCAPD2, NCAPD3, NCAPG2, NCAPH, NCAPH2, NCBP1, NCBP2, NCOA1, NCOA2, NCOA3, NCOA5, NCOA6, NCOR1, NCOR2, NDUFAF5, NDUFAF7, NEK11, NELFA, NELFE, NEUROD1, NEUROG1, NEUROG3, NFAT5, NFATC1, NFATC2, NFATC3, NFATC4, NFE2L1, NFIA, NFKB1, NFKB2, NIBAN2, NIPBL, NKAP, NKAPL, NKX2-2, NKX2-5, NKX3-1, NKX6-1, NNMT, NOC2L, NOC3L, NONO, NOP2, NOP56, NOS1, NOTCH1, NPM1, NPM2, NPM3, NR1H4, NR2C1, NR2E1, NR3C1, NR4A3, NR5A1, NR5A2, NRDE2, NRIP1, NRL, NSD1, NSD2, NSD3, NSUN2, NSUN3, NSUN4, NSUN5, NSUN6, NSUN7, NTMT1, NUCKS1, NUDT21, NUDT5, NUP62, NUP98, NUPR1, OBI1, OGDH, OGT, ONECUT1, ORC1, OTUB1, OTUD4, OVOL2, PABPC1L, PADI1, PADI2, PADI3, PADI4, PADI6, PAF1, PAGR1, PAK1, PARG, PARK7, PARP1, PARP10, PARP9, PATZ1, PAX5, PAX6, PAX7, PAXBP1, PAXIP1, PBRM1, PBX2, PCGF1, PCGF2, PCGF3, PCGF5, PCGF6, PCIF1, PCLAF, PCMT1, PCMTD1, PCMTD2, PCNA, PDX1, PELP1, PEMT, PER1, PER2, PHB, PHC1, PHF1, PHF10, PHF13, PHF19, PHF2, PHF20, PHF21A, PHF23, PHF6, PHF8, PHIP, PICK1, PIH1D1, PIK3CA, PINK1, PIWIL2, PIWIL4, PKN1, PKN2, PKNOX1, PLAC8, PLD6, PLK1, PNMT, POLA1, POLD1, POLE, POLE3, POLG, POLQ, POLR1A, POLR1B, POLR1C, POLR1D, POLR1E, POLR2A, POLR2B, POLR2E, POLR2F, POLR2H, POLR2K, POLR2L, POLR3A, POLR3D, POR, POU1F1, POU4F1, POU4F2, PPARG, PPARGC1A, PPHLN1, PPM1D, PPM1F, PPME1, PPP3CA, PPP5C, PRDM1, PRDM10, PRDM11, PRDM12, PRDM13, PRDM14, PRDM15, PRDM16, PRDM2, PRDM4, PRDM5, PRDM6, PRDM7, PRDM8, PRDM9, PRIMPOL, PRKAA1, PRKAA2, PRKCA, PRKCB, PRKCD, PRKD1, PRKD2, PRKN, PRMT1, PRMT2, PRMT3, PRMT5, PRMT6, PRMT7, PRMT8, PRMT9, PROP1, PRORP, PSME4, PTF1A, PTMA, PWP1, PWWP2A, PWWP3A, PYGO1, PYGO2, RAB3B, RAB3D, RAB6A, RAC1, RAD17, RAD21, RAD21L1, RAD51, RAD9A, RAG1, RAG2, RAMAC, RAMACL, RAN, RARA, RB1, RBBP4, RBBP5, RBBP7, RBL1, RBL2, RBM14, RBM15, RBM15B, RBMX, RBPJ, RBPJL, RCBTB1, RCC1, RCCD1, RCOR1, REC8, REL, RELA, RELB, RERE, RESF1, REST, RFXANK, RIF1, RING1, RIOX1, RIOX2, RIT2, RLF, RNF168, RNF2, RNF20, RNF40, RNF8, RNMT, RPS6KA4, RPS6KA5, RRNAD1, RRP8, RSBN1, RSF1, RTF1, RUNX2, RUVBL1, RUVBL2, RXRA, RYBP, SAFB, SAP30, SAP30L, SART3, SATB1, SATB2, SBNO1, SBNO2, SCMH1, SDR16C5, SET, SETD1A, SETD1B, SETD2, SETD3, SETD4, SETD5, SETD6, SETD7, SETD9, SETDB1, SETDB2, SETMAR, SETSIP, SF3B1, SFMBT1, SFMBT2, SFPQ, SGF29, SHMT2, SIN3A, SIN3B, SIRT1, SIRT2, SIRT3, SIRT6, SIRT7, SIX1, SIX3, SKI, SKIL, SKOR2, SKP1, SLBP, SLC30A9, SMAD2, SMAD3, SMAD4, SMAD6, SMARCA1, SMARCA2, SMARCA4, SMARCA5, SMARCAD1, SMARCB1, SMARCC1, SMARCC2, SMARCD1, SMARCD2, SMARCD3, SMARCE1, SMC1A, SMC3, SMCHD1, SMG5, SMYD1, SMYD2, SMYD3, SMYD4, SMYD5, SNAI2, SNCA, SNRPB, SNRPD3, SNRPE, SNRPF, SNRPG, SNW1, SOX1, SOX10, SOX14, SOX15, SOX2, SOX9, SP1, SP2, SP3, SPHK2, SPI1, SPIN1, SPIN2A, SPIN2B, SPIN3, SPIN4, SPOUT1, SPTY2D1, SRCAP, SREBF1, SRF, SS18L1, SSB, SSBP1, SSRP1, STAG1, STAG2, STAG3, STAT1, STAT3, STAT5B, STPG4, SUDS3, SUPT16H, SUPT3H, SUPT4H1, SUPT5H, SUPT6H, SUPT7L, SUV39H1, SUV39H2, SUZ12, SVEP1, TADA1, TADA2A, TADA2B, TADA3, TAF1, TAF10, TAF12, TAF1A, TAF1B, TAF1C, TAF1D, TAF1L, TAF2, TAF5L, TAF6, TAF6L, TAF7, TAF7L, TAF9, TAL1, TARBP1, TASOR, TBL1X, TBL1XR1, TBL1Y, TBP, TCF21, TCF4, TCF7L1, TCF7L2, TDG, TDRD1, TDRD12, TDRD3, TDRD5, TDRD9, TDRKH, TENT2, TENT4A, TENT4B, TET1, TET2, TET3, TFAM, TFAP2A, TFAP2B, TFAP2C, TFAP4, TFB1M, TFB2M, TGFB1, TGS1, THADA, THRA, THRB, THUMPD2, THUMPD3, TICRR, TLE4, TLK1, TLK2, TNKS, TNP1, TNRC18, TONSL, TOP1, TOP2A, TOP2B, TOX, TOX3, TP53, TP53BP1, TP63, TPMT, TPR, TRAF6, TRDMT1, TRIM16, TRIM24, TRIM27, TRIM28, TRIM37, TRIM66, TRIM68, TRIP12, TRIP4, TRMO, TRMT1, TRMT10A, TRMT10B, TRMT10C, TRMT11, TRMT112, TRMT12, TRMT13, TRMT1L, TRMT2A, TRMT2B, TRMT44, TRMT5, TRMT6, TRMT61A, TRMT61B, TRMT9B, TRMU, TRRAP, TSHZ1, TSHZ2, TSHZ3, TSPYL2, TTC5, TTF1, TTLL12, TWIST1, TWISTNB, TYMS, TYW3, UBE2A, UBE2B, UBE2E1, UBE2N, UBE2T, UBE2U, UBN1, UBR2, UBR5, UBTF, UCN, UHRF1, UHRF1BP1, UHRF2, UIMC1, UPF1, URI1, USF1, USP15, USP16, USP17L2, USP21, USP22, USP3, USP36, USP49, USP51, USP7, USP9X, UTP3, UTY, UXT, VAX1, VAX2, VCPKMT, VCX, VEGFA, VIRMA, VPS72, VRK1, VSX1, WAC, WAPL, WBP2, WBP2NL, WDHD1, WDR13, WDR4, WDR5, WDR5B, WDR61, WDR70, WDR82, WDTC1, WIZ, WRAP53, WRN, WT1, WTAP, XRN1, YAP1, YBX1, YEATS2, YEATS4, YWHAB, YWHAE, ZBTB1, ZBTB7A, ZBTB7B, ZC3H12A, ZC3H13, ZCCHC4, ZEB1, ZFP57, ZIC2, ZKSCAN3, ZMIZ1, ZMIZ2, ZMPSTE24, ZMYND11, ZMYND15,

ZMYND8, ZNF274, ZNF304, ZNF335, ZNF354B, ZNF431, ZNF445, ZNF451, ZNF462, ZNF473, ZNF609, ZNF683, ZNF711, ZNF750, ZNFX1, ZNHIT1, ZNRD1
***Mus musculus* genes**
0610010K14Rik, Abraxas1, Actl6a, Actl6b, Actn4, Actr6, Actr8, Actrt1, Adnp, Aebp2, Aicda, Aire, Ajuba, Ak6, Akap8, Akap8l, Alkbh1, Alkbh2, Alkbh3, Alkbh4, Alkbh5, Alkbh8, Amt, Ankra2, Ankrd1, Ankrd17, Ankrd2, Anp32a, Anp32b, Anp32e, Apbb1, Apbb2, Apex1, Apobec1, Apobec2, Apobec3, App, Aptx, Ar, Arid1a, Arid1b, Arid2, Arid3a, Arid3c, Arid4a, Arid4b, Arid5a, Armt1, Arrb1, Arx, As3mt, Ascl1, Asf1a, Asf1b, Ash1l, Ash2l, Asxl1, Asxl3, Asz1, Atad2, Atad2b, Atf2, Atf5, Atf7ip, Atg5, Atg7, Atm, Atoh1, Atpsckmt, Atrx, Atxn1, Atxn3, Atxn7, Atxn7l3, Aurka, Aurkb, Auts2, Babam1, Babam2, Bag6, Bahcc1, Bahd1, Banp, Bap1, Barx2, Baz1a, Baz1b, Baz2a, BC004004, Bcas3, Bcdin3d, Bcl6, Bcor, Bcorl1, Bend3, Bend6, Bex4, Bhlhe41, Bhmt, Bhmt2, Bmi1, Bmt2, Bptf, Brca1, Brca2, Brcc3, Brd1, Brd2, Brd3, Brd4, Brd7, Brd8, Brd9, Brdt, Brip1, Brms1, Brms1l, Brpf1, Brpf3, Btaf1, Btg1, Btg2, Bud23, Calcoco1, Camkmt, Camta2, Carm1, Carnmt1, Cbfa2t3, Cbll1, Cbx1, Cbx2, Cbx4, Cbx5, Cbx6, Cbx7, Cbx8, Ccna2, Ccnb1, Ccnd1, Ccnt1, Ccnt2, Cdan1, Cdc20, Cdc45, Cdc6, Cdc73, Cdk1, Cdk2, Cdk5, Cdk9, Cdkn2a, Cdt1, Cdyl, Cdyl2, Cebpa, Cebpb, Cebpg, Cecr2, Cenpb, Cenps, Cenpv, Cgas, Chaf1a, Chaf1b, Chd1, Chd1l, Chd2, Chd3, Chd4, Chd5, Chd6, Chd7, Chd8, Chd9, Chek1, Chmp7, Chtop, Cic, Cir1, Cited1, Cited2, Cks1b, Cks1brt, Cks2, Clock, Cmtr1, Cmtr2, Comt, Comtd1, Coprs, Coq3, Coq5, Coq7, Cramp1l, Creb1, Crebbp, Crebzf, Crtc2, Crx, Crxos, Cry1, Csnk2b, Ctbp1, Ctbp2, Ctcf, Ctcfl, Ctnnb1, Ctr9, Ctsl, Cul4b, Cxxc1, Cyp1a1, Cyp1a2, Cyp3a11, Cyp3a13, Cyp3a16, Cyp3a25, Cyp3a41a, Cyp3a41b, Cyp3a44, Cyp3a57, Cyp3a59, Cyp51, D7Ertd443e, Dact1, Dapk3, Daxx, Dcaf1, Dcp2, Ddb1, Ddb2, Ddx1, Ddx11, Ddx20, Ddx4, Dek, Dhx30, Dhx36, Dhx9, Dimt1, Dld, Dlst, Dlx1, Dlx2, Dlx3, Dmap1, Dmrt1, Dmrtc2, Dnajc2, Dnmt1, Dnmt3a, Dnmt3b, Dnmt3c, Dnmt3l, Dot1l, Dpf1, Dpf2, Dpf3, Dph5, Dppa2, Dppa3, Dppa4, Dpy30, Dr1, Drd1, Dtx3l, Dydc1, Dydc2, E2f4, Ebf2, Ecd, Eed, Eef1akmt1, Eef1akmt2, Eef1akmt3, Eef1akmt4, Eef1aknmt, Eef2kmt, Egfr, Egr1, Egr2, Ehmt1, Ehmt2, Eid1, Elk1, Elk4, Elof1, Emg1, Emsy, Eny2, Eomes, Ep300, Ep400, Epas1, Epc1, Epc2, Epop, Ercc1, Ercc3, Ercc4, Ercc6, Erg, Esco2, Esr1, Esr2, Etf1, Etfbkmt, Ets1, Exo1, Exosc10, Exosc4, Eya1, Eya2, Eya3, Eya4, Ezh1, Ezh2, Faap24, Fabp1, Fam172a, Fam173a, Fam50a, Fam50b, Fam98a, Fam98b, Fancm, Fbh1, Fbl, Fbll1, Fbxo11, Fcor, Fdxacb1, Fezf2, Fh1, Fkbp6, Flcn, Fli1, Fmr1, Fos, Fosl2, Foxa1, Foxa2, Foxa3, Foxc1, Foxc2, Foxn4, Foxo3, Foxo4, Foxp1, Foxp3, Fto, Ftsj1, Ftsj3, Fus, Gabpa, Gadd45a, Gamt, Gata1, Gata2, Gata3, Gata4, Gata6, Gatad1, Gcg, Gcm1, Gfi1, Gfi1b, Gk, Gli1, Gli2, Gli3, Glmn, Glyr1, Gm10184, Gm10767, Gm20737, Gm20738, Gm20747, Gm20772, Gm20773, Gm20777, Gm20795, Gm20806, Gm20807, Gm20809, Gm20812, Gm20815, Gm20816, Gm20821, Gm20822, Gm20823, Gm20825, Gm20826, Gm20828, Gm20830, Gm20831, Gm20834, Gm20852, Gm20854, Gm20865, Gm20867, Gm20873, Gm20877, Gm20909, Gm20914, Gm20917, Gm20918, Gm20924, Gm21118, Gm21244, Gm21292, Gm21310, Gm21394, Gm21425, Gm21440, Gm21637, Gm21719, Gm21721, Gm21778, Gm21812, Gm21854, Gm21874, Gm21943, Gm27027, Gm28043, Gm28079, Gm28171, Gm29049, Gm29644, Gm33815, Gm42715, Gm42742, Gm49333, Gm49388, Gm50367, Gm5096, Gm5136, Gm5926, Gmnc, Gmnn, Gnas, Gnmt, Gper1, Gpx1, Gpx4, Grhl1, Grhl2, Grhl3, Grwd1, Gsk3a, Gsk3b, Gspt1, Gtf2b, Gtf2f1, Gtf2h1, Gtf3c4, Gtpbp3, H1f0, H1f1, H1f10, H1f2, H1f3, H1f4, H1f5, H1f7, H1f8, H2ab1, H2ab2, H2ab3, H2ac1, H2ac10, H2ac11, H2ac12, H2ac13, H2ac15, H2ac18, H2ac19, H2ac20, H2ac21, H2ac4, H2ac6, H2ac7, H2ac8, H2aj, H2al1a, H2al1b, H2al1c, H2al1d, H2al1e, H2al1f, H2al1g, H2al1h, H2al1i, H2al1j, H2al1k, H2al1m, H2al1n, H2al1o, H2al2a, H2al2b, H2al2c, H2al3, H2ap, H2aw, H2ax, H2az1, H2az2, H2bc1, H3c13, H3c14, H3c15, H3c2, H3c3, H3c4, H3c6, H3c7, H3f3a, H3f3b, Haspin, Hat1, Hcfc1,

Hcfc2, Hdac1, Hdac10, Hdac11, Hdac2, Hdac3, Hdac4, Hdac5, Hdac6, Hdac7, Hdac8, Hdac9, Hdgfl3, Hells, Hemk1, Henmt1, Hes1, Hes5, Hesx1, Hey2, Hhex, Hic1, Hif1a, Hils1, Hinfp, Hipk4, Hira, Hist1h2an, Hist1h2ao, Hist1h2ap, Hjurp, Hlcs, Hltf, Hmbox1, Hmg20a, Hmg20b, Hmga1, Hmga1b, Hmga2, Hmgb1, Hmgb2, Hmgb3, Hmgb4, Hmgn1, Hmgn3, Hmgn5, Hnf1a, Hnf4a, Hnmt, Hnrnpd, Hnrnpu, Hopx, Hoxa10, Hoxc13, Hoxd10, Hoxd13, Hp1bp3, Hpf1, Hr, Hsd17b10, Hsf1, Hsf4, Hsp90aa1, Hsp90ab1, Hspa1a, Huwe1, Icmt, Ift74, Igf2, Ikzf3, Ilf3, Incenp, Ing1, Ing2, Ing3, Ing4, Ing5, Inmt, Ino80, Ino80b, Ino80c, Ino80e, Insm1, Ipo7, Irf4, Isl1, Iws1, Jade1, Jade2, Jade3, Jak2, Jarid2, Jdp2, Jmjd1c, Jmjd6, Jmjd7, Jun, Kansl1, Kansl1l, Kansl2, Kansl3, Kat14, Kat2a, Kat2b, Kat5, Kat6a, Kat6b, Kat7, Kat8, Kctd21, Kdm1a, Kdm1b, Kdm2a, Kdm2b, Kdm3a, Kdm4a, Kdm4b, Kdm4c, Kdm4d, Kdm5a, Kdm5b, Kdm5c, Kdm5d, Kdm6a, Kdm6b, Kdm7a, Kdm8, Klf14, Klf2, Klf4, Klhdc3, Kmt2a, Kmt2b, Kmt2c, Kmt2d, Kmt2e, Kmt5a, Kmt5b, Kmt5c, Kpna2, Kpna7, L3mbtl1, L3mbtl2, L3mbtl3, Larp7, Lcmt1, Lcmt2, Lcor, Ldb1, Lef1, Lemd2, Leo1, Lhx2, Lif, Lmna, Lmo2, Loxl2, Lrrk2, Lrwd1, Lsm1, Lsm10, M1ap, Macroh2a1, Macroh2a2, Mael, Map1s, Map3k12, Map3k7, Mapk15, Mapk3, Mapk8, Mbd2, Mbd3, Mbd3l1, Mbd3l2, Mbd5, Mbd6, Mbip, Mbtd1, Mcm2, Mcm3ap, Mcm8, Mcm9, Mcmbp, Mcrs1, Meaf6, Mecom, Mecp2, Med1, Med12, Med24, Med25, Mef2a, Mef2b, Mef2c, Mef2d, Meiob, Meis1, Meis3, Men1, Meox1, Mepce, Mettl1, Mettl11b, Mettl14, Mettl15, Mettl16, Mettl17, Mettl18, Mettl2, Mettl21a, Mettl21c, Mettl21e, Mettl22, Mettl23, Mettl24, Mettl3, Mettl4, Mettl5, Mettl6, Mettl7a1, Mettl7a2, Mettl7b, Mettl8, Mgmt, Mier1, Mier2, Mier3, Mir744, Mis18a, Mitf, Mki67, Mkrn1, Mlh1, Mlh3, Mllt10, Mllt3, Mllt6, Mmachc, Mnt, Morc1, Morc2a, Morf4l1, Morf4l2, Mos, Mphosph8, Mrgbp, Mrm1, Mrm2, Mrm3, Mrnip, Msgn1, Msh2, Msh6, Msl1, Msl2, Msl3, Msl3l2, Mst1, Msx3, Mt3, Mta1, Mta2, Mta3, Mtap, Mtf1, Mtf2, Mthfr, Mtpap, Mtr, Myb, Myc, Myocd, Myod1, Myog, Mysm1, N6amt1, Naa40, Naa50, Naa60, Nacc2, Nanog, Nap1l2, Nasp, Nat8f6, Nat8f7, Ncapd2, Ncapd3, Ncapg2, Ncaph, Ncaph2, Ncl, Ncoa1, Ncoa2, Ncoa3, Ncoa5, Ncor1, Ncor2, Ndufaf5, Ndufaf7, Nek11, Nelfa, Nelfe, Neurod1, Neurog1, Neurog3, Nfat5, Nfatc2, Nfatc4, Nfe2l1, Nfia, Nfkb1, Nfkb2, Nipbl, Nkap, Nkapl, Nkx2-2, Nkx2-5, Nkx3-1, Nkx6-1, Nnmt, Noc2l, Noc3l, Nono, Nop2, Nop56, Nos1, Notch1, Npm1, Npm2, Npm3, Nr1h4, Nr2c1, Nr2e1, Nr4a3, Nr5a1, Nr5a2, Nrde2, Nrip1, Nrl, Nsd1, Nsd2, Nsd3, Nsun2, Nsun3, Nsun4, Nsun5, Nsun6, Nsun7, Ntmt1, Nucks1, Nudt21, Nudt5, Nup153, Nup98, Nupr1, Oga, Ogdh, Ogt, Onecut1, Orc1, Otub1, Otud4, Ovol2, Pabpc1l, Padi1, Padi2, Padi3, Padi4, Padi6, Paf1, Pagr1a, Pagr1b, Pak1, Parg, Park7, Parp1, Parp10, Parp9, Patz1, Pax3, Pax5, Pax6, Pax7, Paxbp1, Paxip1, Pbrm1, Pbx2, Pcgf1, Pcgf2, Pcgf3, Pcgf5, Pcgf6, Pcif1, Pclaf, Pcmt1, Pcmtd1, Pcmtd2, Pcna, Pdx1, Pelp1, Pemt, Per1, Phb, Phc1, Phf1, Phf13, Phf19, Phf2, Phf20, Phf21a, Phf23, Phf6, Phf8, Phip, Pih1d1, Pik3ca, Pim3, Pink1, Pitx2, Piwil2, Piwil4, Pkn1, Pkn2, Pknox1, Pla2g4a, Plac8, Pld6, Plk1, Pml, Pnmt, Pola1, Pold1, Pole, Pole3, Polg, Polq, Polr1a, Polr2a, Polr2b, Polr3a, Polr3d, Por, Pou1f1, Pou2f1, Pou4f1, Pou4f2, Pou5f1, Ppargc1a, Ppm1d, Ppm1f, Ppme1, Ppp3ca, Ppp5c, Prdm1, Prdm10, Prdm11, Prdm12, Prdm13, Prdm14, Prdm15, Prdm16, Prdm2, Prdm4, Prdm5, Prdm6, Prdm9, Primpol, Prkaa1, Prkaa2, Prkca, Prkcb, Prkcd, Prkd1, Prkd2, Prkn, Prmt1, Prmt2, Prmt3, Prmt5, Prmt6, Prmt7, Prmt8, Prmt9, Prop1, Psip1, Psme4, Ptf1a, Ptma, Pwp1, Pwwp2a, Pwwp3a, Pygo1, Pygo2, Rab3b, Rab3d, Rab6a, Rac1, Rad17, Rad21, Rad21l, Rad51, Rad9a, Rag1, Rag2, Ramac, Rara, Rb1, Rbbp4, Rbbp5, Rbbp7, Rbl1, Rbl2, Rbm14, Rbm15, Rbm15b, Rbmx, Rbmxl1, Rbpj, Rbpjl, Rcc1, Rccd1, Rcor1, Rec8, Rela, Relb, Rere, Resf1, Rest, Rfxank, Rif1, Ring1, Riox1, Riox2, Rit2, Rlf, Rnf168, Rnf2, Rnf20, Rnf219, Rnf40, Rnf8, Rnmt, Rpa1, Rps6ka4, Rps6ka5, Rrp8, Rsbn1, Rsf1, Rsl1, Rtf1, Runx2, Runx3, Ruvbl1, Ruvbl2, Rxra, Rybp, Safb, Sap30, Sap30l, Sart3, Satb1, Satb2, Sbno2, Scmh1, Scml2, Sdr16c5, Senp2, Setd1a, Setd1b, Setd2, Setd3, Setd4, Setd5, Setd6, Setd7, Setdb1, Setdb2, Setmar, Sfmbt1, Sfmbt2, Sfpq, Sgf29, Shmt2, Sik1, Sin3a, Sin3b, Sirt1, Sirt2, Sirt3, Sirt6, Sirt7, Six1, Six3, Skil, Skor2, Skp1a, Slbp, Slc30a9, Slk, Smad2, Smad3, Smad4, Smad6, Smarca1, Smarca2, Smarca4, Smarca5, Smarcad1, Smarcb1, Smarcc1, Smarcc2, Smarcd1, Smarcd2, Smarcd3, Smarce1, Smc1a, Smc3, Smchd1, Smg5, Smyd1, Smyd2,

Smyd3, Smyd4, Smyd5, Snai2, Snca, Snrpb, Snrpd3, Sox10, Sox14, Sox15, Sox9, Sp1, Sp2, Sp3, Sphk2, Spi1, Spin1, Spin2c, Spin2d, Spin2e, Spin2f, Spin2g, Spin4, Spout1, Spty2d1, Srcap, Srebf1, Srebf2, Srf, Ss18l1, Ssbp1, Ssrp1, Ssty2, Stag1, Stag2, Stag3, Stat1, Stat3, Stat5b, Stpg4, Suds3, Supt3, Supt4a, Supt5, Supt6, Supt7l, Suv39h1, Suv39h2, Suz12, Svep1, Tada1, Tada2a, Tada2b, Tada3, Taf1, Taf10, Taf12, Taf5l, Taf6, Taf6l, Taf7, Taf7l, Taf9, Tal1, Tarbp1, Tasor, Tbl1x, Tbl1xr1, Tcf21, Tcf3, Tcf4, Tcf7l2, Tdg, Tdrd1, Tdrd12, Tdrd3, Tdrd5, Tdrd9, Tdrkh, Tent2, Tent4a, Tent4b, Tert, Tet1, Tet2, Tet3, Tfam, Tfap2b, Tfap2c, Tfap4, Tfb1m, Tfb2m, Tgfb1, Tgif1, Tgs1, Thada, Thra, Thrb, Thumpd2, Thumpd3, Ticrr, Tle1, Tle4, Tlk1, Tlk2, Tmem29, Tnks, Tnp1, Tnrc18, Tomt, Tonsl, Top1, Top2a, Top2b, Tox, Tox3, Tpr, Traf6, Trdmt1, Trim16, Trim24, Trim27, Trim28, Trim37, Trim66, Trim68, Trip12, Trip4, Trmo, Trmt1, Trmt10a, Trmt10b, Trmt10c, Trmt11, Trmt112, Trmt12, Trmt13, Trmt1l, Trmt2a, Trmt2b, Trmt44, Trmt5, Trmt6, Trmt61a, Trmt9b, Trp53, Trp53bp1, Trp63, Trrap, Tshz1, Tshz2, Tshz3, Tspyl2, Ttc5, Ttf1, Ttll12, Twist1, Tyms, Tyw3, Ube2a, Ube2b, Ube2e1, Ube2n, Ube2t, Ubn1, Ubr2, Ubr5, Ubtf, Ucn, Uhrf1, Uhrf1bp1, Uhrf2, Uimc1, Upf1, Uri1, Usf1, Usp15, Usp16, Usp22, Usp3, Usp36, Usp49, Usp51, Usp7, Usp9x, Utp3, Uty, Uxt, Vax1, Vax2, Vcpkmt, Vegfa, Virma, Vps72, Vrk1, Vsx1, Wac, Wapl, Wbp2, Wbp2nl, Wdhd1, Wdr13, Wdr4, Wdr5, Wdr5b, Wdr61, Wdr82, Wdtc1, Wiz, Wrap53, Wrn, Wtap, Xbp1, Xrn1, Yap1, Ybx1, Yeats2, Yeats4, Ywhab, Ywhae, Zbtb1, Zbtb7a, Zbtb7b, Zc3h12a, Zc3h13, Zcchc4, Zeb1, Zfp143, Zfp335, Zfp354b, Zfp386, Zfp445, Zfp451, Zfp462, Zfp57, Zfp609, Zfp683, Zfp750, Zfp932, Zfp936, Zmiz1, Zmiz2, Zmpste24, Zmynd11, Zmynd15, Zmynd8, Znfx1, Znhit1

Supplementary Table 4. Differential gene expression for epigenetics related genes in murine embryonic stem-cells (mESC) assays

Gene Name	Gene Ontology	Gene Definition	Valproic Acid		Carbamazepine		Phenytoin		Methotrexate		Warfarin	
			logFC	Adj P-Value	logFC	Adj P-Value	logFC	Adj P-Value	logFC	Adj P-Value	logFC	Adj P-Value
Dmrt1	GO:0003682	chromatin binding	0.980	0.000	-1.493	0.000	0.623	0.060	-1.496	0.000	-1.531	0.000
Eomes	GO:0003682	chromatin binding	-2.655	0.000	3.715	0.000	-2.146	0.003	3.398	0.000	3.422	0.000
Foxa2	GO:0003682	chromatin binding	-0.915	0.001	1.482	0.000	-1.129	0.002	1.961	0.000	1.851	0.000
Hhex	GO:0003682	chromatin binding	-1.114	0.000	1.078	0.000	-0.871	0.002	1.584	0.000	1.572	0.000
Isl1	GO:0003682	chromatin binding	-0.934	0.001	1.536	0.000	-0.997	0.004	1.996	0.000	2.046	0.000
Lef1	GO:0003682	chromatin binding	-1.889	0.000	1.468	0.001	-1.331	0.001	1.643	0.001	1.641	0.002
Mecom	GO:0000118	histone deacetylase complex	1.123	0.000	-1.929	0.000	1.114	0.005	-1.655	0.001	-1.911	0.000
Meis1	GO:0003682	chromatin binding	1.690	0.000	-2.675	0.000	1.432	0.006	-2.314	0.000	-2.336	0.001
Pax3	GO:0003682	chromatin binding	0.989	0.001	-1.840	0.000	1.196	0.002	-1.830	0.000	-1.839	0.000
Pax6	GO:0003682	chromatin binding	2.294	0.000	-3.319	0.000	1.983	0.003	-3.103	0.000	-3.416	0.000
Rec8	GO:0003682	chromatin binding	1.506	0.000	-1.291	0.000	0.583	0.027	-1.144	0.000	-0.987	0.004
Tgfb1	GO:0031065	positive regulation of histone deacetylation	-0.656	0.003	1.281	0.000	-0.922	0.001	1.606	0.000	1.717	0.000
Tshz1	GO:0003682	chromatin binding	2.354	0.000	-3.273	0.000	2.118	0.003	-2.851	0.001	-2.888	0.002

Supplementary Table 5. Differential gene expression for epigenetics related genes in human embryonic stem cells (hESC) assays

Drug	Gene Name	Gene Ontology	Gene Definition	logFC	Adj P-Value
Valproic Acid	CTCFL	GO:0016571	histone methylation	3.629465075	3.01E-34
	NEUROD1	GO:0003682	chromatin binding	-1.936291357	0.005733283
	PADI3	GO:0036414	histone citrullination	1.834979456	3.04E-17
	PRKCB	GO:0003682	chromatin binding	3.518753762	8.51E-26
	PPARGC1A	GO:0031490	chromatin DNA binding	1.911997754	1.81E-23
	ASMTL	GO:0032259	methylation	1.586826695	1.53E-19
	TSHZ2	GO:0003682	chromatin binding	1.716959415	7.74E-21
	SNCA	GO:0042393	histone binding	2.107120519	1.13E-24
	EYA4	GO:0016576	histone dephosphorylation	2.986393142	2.64E-23
Carbamazepine	HESX1	GO:0003682	chromatin binding	-2.947542562	2.45E-11
	APOBEC3B	GO:0080111	DNA demethylation	-1.586545164	1.04608E-08

Supplementary Table 6. Characteristics of the mouse embryonic stem-cell (mESC) samples included in WGCNA analysis (retrieved from E-TABM-1205 and E-TABM-1216 studies)

Source Name	cell	concentration	exposure	time
PT101_(Mouse430_2)	ES-D3 - mESC	3	carbamazepine	4-day differentiated
PT_102_(Mouse430_2)	ES-D3 - mESC	3	carbamazepine	4-day differentiated
PT_104_(Mouse430_2)	ES-D3 - mESC	3	carbamazepine	4-day differentiated
PT105_(Mouse430_2)	ES-D3 - mESC	3	carbamazepine	4-day differentiated
PT_106_(Mouse430_2)	ES-D3 - mESC	3	carbamazepine	4-day differentiated
PT_108_(Mouse430_2)	ES-D3 - mESC	3	carbamazepine	4-day differentiated
PT109_(Mouse430_2)	ES-D3 - mESC	3	carbamazepine	4-day differentiated
PT110_(Mouse430_2)	ES-D3 - mESC	3	carbamazepine	4-day differentiated
PT111_(Mouse430_2)	ES-D3 - mESC	100	carbamazepine	4-day differentiated
PT_112_(Mouse430_2)	ES-D3 - mESC	100	carbamazepine	4-day differentiated
PT113_(Mouse430_2)	ES-D3 - mESC	100	carbamazepine	4-day differentiated
PT114_(Mouse430_2)	ES-D3 - mESC	100	carbamazepine	4-day differentiated
PT_115_(Mouse430_2)	ES-D3 - mESC	100	carbamazepine	4-day differentiated
PT_117_(Mouse430_2)	ES-D3 - mESC	100	carbamazepine	4-day differentiated
PT118_(Mouse430_2)	ES-D3 - mESC	100	carbamazepine	4-day differentiated
PT_119_(Mouse430_2)	ES-D3 - mESC	100	carbamazepine	4-day differentiated
PT121_(Mouse430_2)	ES-D3 - mESC	1	phenytoin	4-day differentiated
PT123_(Mouse430_2)	ES-D3 - mESC	1	phenytoin	4-day differentiated
PT125_(Mouse430_2)	ES-D3 - mESC	1	phenytoin	4-day differentiated
PT_126_(Mouse430_2)	ES-D3 - mESC	1	phenytoin	4-day differentiated
PT127_(Mouse430_2)	ES-D3 - mESC	1	phenytoin	4-day differentiated
PT_128_(Mouse430_2)	ES-D3 - mESC	1	phenytoin	4-day differentiated
PT_129_(Mouse430_2)	ES-D3 - mESC	1	phenytoin	4-day differentiated
PT_130_(Mouse430_2)	ES-D3 - mESC	1	phenytoin	4-day differentiated
PT131_(Mouse430_2)	ES-D3 - mESC	30	phenytoin	4-day differentiated
PT_132_(Mouse430_2)	ES-D3 - mESC	30	phenytoin	4-day differentiated
PT_134_(Mouse430_2)	ES-D3 - mESC	30	phenytoin	4-day differentiated
PT135_(Mouse430_2)	ES-D3 - mESC	30	phenytoin	4-day differentiated
PT_136_(Mouse430_2)	ES-D3 - mESC	30	phenytoin	4-day differentiated
PT138_(Mouse430_2)	ES-D3 - mESC	30	phenytoin	4-day differentiated
PT139_(Mouse430_2)	ES-D3 - mESC	30	phenytoin	4-day differentiated
PT_140_(Mouse430_2)	ES-D3 - mESC	30	phenytoin	4-day differentiated
COND4_1	ES-D3 - mESC	0	none	4-day differentiated
COND4_3n	ES-D3 - mESC	0	none	4-day differentiated
COND4_4	ES-D3 - mESC	0	none	4-day differentiated
COND4_5n	ES-D3 - mESC	0	none	4-day differentiated
COND4_7	ES-D3 - mESC	0	none	4-day differentiated
VPA15_1	ES-D3 - mESC	15	valproic acid	4-day differentiated
VPA15_2	ES-D3 - mESC	15	valproic acid	4-day differentiated
VPA15_3	ES-D3 - mESC	15	valproic acid	4-day differentiated

VPA15_4	ES-D3 - mESC	15	valproic acid	4-day differentiated
VPA15_5	ES-D3 - mESC	15	valproic acid	4-day differentiated
VPA15_6	ES-D3 - mESC	15	valproic acid	4-day differentiated
VPA15_7	ES-D3 - mESC	15	valproic acid	4-day differentiated
VPA15_8	ES-D3 - mESC	15	valproic acid	4-day differentiated
VPA60_1	ES-D3 - mESC	60	valproic acid	4-day differentiated
VPA60_2	ES-D3 - mESC	60	valproic acid	4-day differentiated
VPA60_3	ES-D3 - mESC	60	valproic acid	4-day differentiated
VPA60_5	ES-D3 - mESC	60	valproic acid	4-day differentiated
VPA60_6	ES-D3 - mESC	60	valproic acid	4-day differentiated
VPA60_7	ES-D3 - mESC	60	valproic acid	4-day differentiated
VPA60_8	ES-D3 - mESC	60	valproic acid	4-day differentiated
VPA60_10	ES-D3 - mESC	60	valproic acid	4-day differentiated
VPA250_1	ES-D3 - mESC	250	valproic acid	4-day differentiated
VPA250_2	ES-D3 - mESC	250	valproic acid	4-day differentiated
VPA250_3	ES-D3 - mESC	250	valproic acid	4-day differentiated
VPA250_4	ES-D3 - mESC	250	valproic acid	4-day differentiated
VPA250_5	ES-D3 - mESC	250	valproic acid	4-day differentiated
VPA250_6	ES-D3 - mESC	250	valproic acid	4-day differentiated
VPA250_7	ES-D3 - mESC	250	valproic acid	4-day differentiated
VPA250_8	ES-D3 - mESC	250	valproic acid	4-day differentiated
VPA1000_1	ES-D3 - mESC	1000	valproic acid	4-day differentiated
VPA1000_2	ES-D3 - mESC	1000	valproic acid	4-day differentiated
VPA1000_3	ES-D3 - mESC	1000	valproic acid	4-day differentiated
VPA1000_4	ES-D3 - mESC	1000	valproic acid	4-day differentiated
VPA1000_5	ES-D3 - mESC	1000	valproic acid	4-day differentiated
VPA1000_6	ES-D3 - mESC	1000	valproic acid	4-day differentiated
VPA1000_8	ES-D3 - mESC	1000	valproic acid	4-day differentiated
VPA1000_10	ES-D3 - mESC	1000	valproic acid	4-day differentiated

Supplementary Table 7. Complete list of genes selected in WGCNA and differential gene expression analysis

**Valproic Acid**
AC106886.6, ACTL6B, ANP32E, APOBEC3B, APOBEC3G, ARID1A, ASMTL, ASXL1, ATF5, AURKB, AUTS2, BAG6, BAZ2B, BCAS3, BHMT, BMI1, BRD1, BRD3, BRD7, BRIP1, BRPF3, C17orf49, CALCOCO1, CARM1, CBX2, CBX3, CBX7, CCNB1, CCNT1, CCNT2, CEBPA, CHD3, CHD6, CHD7, CIC, CITED1, CITED2, COMMD3-BMI1, CRAMP1, CREB1, CREBBP, CREBZF, CRY1, CTCFL, CTNNB1, CUL4B, CYP1A1, CYP51A1, DAPK3, DDX4, DHX30, DNAJC2, DNMT3A, DPF1, DTX3L, E2F4, EGFR, EGR1, EGR2, EHMT2, EID1, ELK4, EOMES, EP400, EPC1, EPC2, EPOP, ERG, ETF1, ETS1, EYA4, FBXO11, FOSL2, FOXA2, FUS, GABPA, GAMT, GATA3, GATA6, GLI3, GLYR1, GNAS, GRHL1, GRHL2, GSPT1, GTPBP3, H1-10, H1-4, HCFC1, HDAC7, HDAC9, HESX1, HEY2, HINFP, HMBOX1, HMG20B, HMGB3, HNF4A, HNMT, HNRNPC, HSF1, HUWE1, IKZF1, ING3, ING5, INO80B, INO80B-WBP1, INO80E, IRF4, ISL1, IWS1, JADE1, JMJD1C, KANSL1, KAT2A, KAT6A, KCTD21, KDM2A, KDM2B, KDM4A, KDM4B, KDM5C, KDM6A, KLHDC3, KMT2A, KMT2D, KMT5A, L3MBTL2, L3MBTL4, LDB1, LEF1, LMNA, LOXL2, MAP3K7, MAPT, MAT1A, MCM9, MECOM, MECP2, MED12, MEIS1, MEIS3, MEN1, METTL1, METTL7B, MIER3, MLH3, MSL1, MTA2, MTA3, MYB, MYC, MYOCD, NCOA1, NCOR1, NCOR2, NEUROD1, NFATC1, NFATC2, NFATC4, NFIA, NFKB2, NIPBL, NKX3-1, NOS1, NPM2, NR2C1, NR4A3, NSD1, NSUN7, NUCKS1, OGDH, OGT, PADI2, PADI3, PAK1, PARP9, PATZ1, PAX3, PAX5, PAX6, PBRM1, PCGF1, PCGF2, PCGF5, PCIF1, PCMTD2, PER2, PHF2, PHF8, PHIP, PIK3CA, PKNOX1, POLQ, POR, PPARGC1A, PRDM10, PRDM5, PRKAA1, PRKAA2, PRKCA, PRKCB, PRKCD, RAB3B, RARA, RBL1, REC8, RUNX2, RUVBL2, SATB2, SETMAR, SFMBT2, SIRT2, SIRT6, SKI, SMARCA2, SMARCA4, SMARCD1, SMARCD3, SMC1A, SMYD3, SNCA, SNRPD3, SOX10, SOX9, SP2, SPHK2, SPIN3, SPIN4, SRCAP, STAG2, STAT5B, SUPT6H, SUZ12, TADA2B, TAF1A, TAL1, TCF4, TCF7L2, TDRD12, TET1, TET3, TGFB1, TGS1, THRB, TLK1, TLK2, TNP1, TNRC18, TOP1, TP53, TPMT, TRDMT1, TRIM27, TRMT2B, TSHZ1, TSHZ2, TTC5, TWISTNB, UHRF1BP1, UPF1, USP3, USP36, USP7, USP9X, VEGFA, VPS72, WBP2, WDR4, WDTC1, WIZ, YWHAE, ZBTB1, ZMIZ1, ZMYND11, ZMYND8, ZNF750, ZNHIT1
**Carbamazepine**
APOBEC3B, ATM, BHMT2, BRCA1, BRD8, CBX5, CHD2, CTCFL, CTNNB1, ELK4, EOMES, ETS1, FLI1, FOXA2, FOXO3, FUS, GRHL2, GRHL3, GSPT1, HESX1, IFI16, ISL1, LEF1, MECOM, MEIS1, MTA3, NUCKS1, OVOL2, PATZ1, PAX3, PAX6, POLR2E, PRKD1, REC8, SMARCA2, SP1, TAL1, TGFB1, TSHZ1, YWHAE
**Phenytoin**
BRCC3, BRD8, BRDT, CHEK1, CYP1A1, DMRT1, DNMT3L, EGFR, EOMES, EYA4, EZH1, FEZF2, FOXC1, GCM1, HES5, JMJD1C, KLF2, KPNA7, MITF, MORC1, NEUROD1, NR5A2, NSUN6, NUPR1, PAX6, PKNOX1, PRDM14, PRKD2, PTF1A, SFMBT2, SSBP1, TDRD12, TET2, TRIP4, TSHZ1

Supplementary Table 8. Association of candidate epigenetics genes and HPO phenotypes for phenytoin and valproic acid embryopathies

**Fetal Hydantoin Syndrome**	EYA4	FOXC1	MITF	NEUROD1	PAX6	PTF1A	TET2	TRIP4	TSHZ1
Hearing abnormality	X	X	X		X		X		X
Everted lower lip vermilion		X			X				
Hypertelorism		X						X	
Strabismus		X	X		X				
Ptosis			X		X				
Intrauterine growth retardation				X					
Cryptorchidism					X			X	
Short stature					X				
Global developmental delay					X			X	
Microcephaly					X	X			
Abnormality of the pinna						X			

**Fetal Valproate Syndrome**	NFIA	MECP2	STAG2	KDM5C	GATA6	FBXO11	HESX1	NIPBL	CHD3	DHX30	KDM6A	SMC1A	MED12	HCFC1	USP9X	HUWE1	SOX9	CUL4B	SATB2	NSD1	CREBBP	CTNNB1	KANSL1	WDR4	PCGF2	POR	GLI3	KAT6A	SRCAP	CITED2	PHIP	CHD7	BRIP1	PIK3CA	SMARCA4	SKI	SUZ12	KMT2A	RUNX2	MAP3K7	PAX3	SMARCA2	TLK2	LMNA	AUTS2	ARID1A
Narrow mouth	X	X											X			X			X		X				X							X		X		X					X		X	X	X	
Downturned corners of mouth								X				X												X				X	X									X								
Short nose	X							X				X															X				X							X			X					
Epicanthus		X	X						X	X	X	X	X			X		X			X		X	X				X			X	X	X	X	X	X		X					X		X	X
Thin vermilion border	X	X	X	X	X			X				X	X	X	X	X			X			X			X			X	X	X	X							X	X			X		X		
Long philtrum		X	X					X				X	X	X	X	X			X	X		X				X					X					X	X	X		X		X				
Depressed nasal ridge						X	X										X			X							X								X	X					X					X
Omphalocele																																X														
