# Supplementary material for: Anticonvulsants and Chromatin-Genes Expression: A Systems Biology Investigation
Source: Front Neurosci. 2020 Nov 25;14:591196. doi: 10.3389/fnins.2020.591196 (PMC7732676; doi:10.3389/fnins.2020.591196)
Supplement: Supplementary file 2 [file Data_Sheet_2.pdf]

FIGURE LEGENDS

Figure 1: Diagram demonstrating the gene filters for each bioinformatic and systems biology analysis performed.

Figure 2: (A) Network for the candidate genes encountered for valproic acid (blue), compared to the genes obtained in carbamazepine and phenytoin evaluations. (B) Network statistics for valproic acid selected genes. Warm colors: high closeness centrality score. Node size: big nodes for genes with high betweenness centrality score.

Figure 3: Gene ontologies enrichment for carbamazepine (A), phenytoin (B) and valproic acid (C) selected genes, and Reactome database enriched pathways for carbamazepine (D), phenytoin (E) and valproic acid (F) drugs.

Figure 4: (A) Comparison of valproic acid candidate genes obtained in the present study (red) and former HPO database registered genes for Fetal Valproate Syndrome (green). Common genes between both strategies are represented in blue, which can be better visualized in the zoom in (Figure 4B).
